# Metabolomics reveal distinct molecular pathways associated with future risk of Crohn’s Disease

**DOI:** 10.1080/19490976.2025.2546998

**Published:** 2025-09-05

**Authors:** Mingyue Xue, Sun-Ho Lee, Jingchen Shao, Haim Leibovitzh, Hien Q. Huynh, Anne M. Griffiths, Dan Turner, Karen L. Madsen, Paul Moayyedi, A. Hillary Steinhart, Mark S. Silverberg, Colette Deslandres, Alain Bitton, David R. Mack, Kevan Jacobson, Mark J. Ropeleski, Maria Cino, Guy Aumais, Charles N. Bernstein, Remo Panaccione, Brian Bressler, Osvaldo Espin-Garcia, Wei Xu, Williams Turpin, Kenneth Croitoru, on behalf of the Crohn’s and Colitis Canada Genetics Environment Microbial Project Research Consortium

**Affiliations:** a Zane Cohen Centre for Digestive Diseases, Mount Sinai Hospital, Toronto, Ontario, Canada; b Division of Gastroenterology & Hepatology, Temerty Faculty of Medicine, University of Toronto, Toronto, Ontario, Canada; c Division of Mathematics and Statistics, Faculty of Art & Science, University of Toronto, Toronto, Ontario, Canada; d Division of Gastroenterology and Nutrition, Department of Pediatrics, Faculty of Medicine and Dentistry, University of Alberta, Edmonton, Alberta, Canada; e IBD Center, The Hospital for Sick Children, Department of Pediatrics, Faculty of Medicine, University of Toronto, Toronto, Ontario, Canada; f The Juliet Keidan Institute of Pediatric Gastroenterology and Nutrition, The Eisenberg R&D Authority, Shaare Zedek Medical Center, The Hebrew University of Jerusalem, Jerusalem, Israel; g Center of Excellence for Gastrointestinal Inflammation and Immunity Research, University of Alberta, Edmonton, Alberta, Canada; h Department of Medicine, McMaster University Farncombe Family Digestive Health Research Institute, Hamilton, Ontario, Canada; i Division of Gastroenterology, Hepatology and Nutrition, Department of Pediatrics, Sainte-Justine Hospital, University of Montreal, Montreal, Quebec, Canada; j Division of Gastroenterology and Hepatology, McGill University Health Centre, Montreal, Quebec, Canada; k Division of Gastroenterology, Hepatology & Nutrition, Children’s Hospital of Eastern Ontario and University of Ottawa, Ottawa, Ontario, Canada; l Canadian Gastro-Intestinal Epidemiology Consortium, Canada, British Columbia Children’s Hospital, British Columbia Children’s Hospital Research Institute, University of British Columbia, Vancouver, BC, Canada; m Department of Medicine, Queen’s University, Kingston, ON, Canada; n Department of Medicine, Division of Gastroenterology, University of Toronto, Toronto, Ontario, Canada; o Hôpital Maisonneuve-Rosemont, Department of Medicine, Montreal University, Montreal, Quebec, Canada; p Inflammatory Bowel Disease Clinical and Research Centre and Department of Internal Medicine, Max Rady College of Medicine, Rady Faculty of Health Sciences, University of Manitoba, Winnipeg, MB, Canada; q Inflammatory Bowel Disease Clinic, Division of Gastroenterology and Hepatology of Gastroenterology, University of Calgary, Calgary, Alberta, Canada; r Department of Medicine, Division of Gastroenterology, St. Paul’s Hospital, University of British Columbia, Vancouver, BC, Canada; s Biostatistics Department, Princess Margaret Cancer Centre, University Health Network, Toronto, Ontario, Canada; t Division of Biostatistics, Dalla Lana School of Public Health, University of Toronto, Toronto, Ontario, Canada; u Department of Nutritional Sciences, Temerty Faculty of Medicine at the University of Toronto, Toronto, Ontario, Canada

**Keywords:** Inflammatory bowel disease, risk biomarkers, gut barrier function, *Ruminococcus torques*

## Abstract

Host – microbiome interactions are central to Crohn’sdisease (CD) pathogenesis; yet the early metabolic alterations that precededisease onset remain poorly defined. To explore preclinical metabolicsignatures of CD, we analyzed baseline serum metabolomic profiles in a nestedcase-control study within the Crohn’s and Colitis Canada – Genetics, Environment, Microbiome (CCC-GEM) Project, a prospective cohort of 5,122 healthyfirst-degree relatives (FDRs) of CD patients. We included 78 individuals wholater developed CD and 311 matched FDRs who remained disease-free. In an untargetedassessment of metabolomic data, we identified 63 metabolites significantlyassociated with future CD risk. Integrative analyses further identifiedmultiple associations between CD-related metabolites and proteomic markers, gutmicrobiome composition, antimicrobial antibody, fecal calprotectin andC-reactive protein. Quinolinate, a tryptophan catabolite, was elevated inindividuals who later developed CD and showed strong positive correlations withC-reactive protein, fecal calprotectin, and C-X-C motif chemokine ligand 9 (CXCL9).In contrast, higher levels of ascorbate and isocitrate were associated withreduced CD risk and were negatively correlated with C-reactive protein and CD-associated proteins.These findings identify several distinct molecular pathways that contribute toCD pathogenesis.

## Introduction

Crohn’s disease (CD) is a debilitating inflammatory bowel disease (IBD) with a rising global incidence.^1^ While the cause(s) of CD is unclear, it is thought to involve a complex interplay between genetic susceptibility, changes in the gut microbiota, and environmental factors.^2–5^ To date, there is no cure for CD,^6–8^ and while existing treatments can alleviate symptoms, their prolonged usage often fails to alter the disease course, often leading to the need for surgery. Therefore, it is clear that novel therapeutic targets that modify the disease course are needed.

Metabolomics analysis of serum offers insights into the end products of both the host and microbial metabolism and their interactions. These reflect the complex interplay between the host genetics, the environment, and the physiological responses that might be associated with disease pathogenesis.^9^ While metabolic changes in established IBD have been reported in several studies, these were limited to cross-sectional studies comparing IBD patients to healthy controls. Such studies are confounded by many factors, including the direct impact of inflammation on metabolic changes. Therefore, it is difficult to distinguish whether metabolic signatures are etiological contributors to CD or merely consequences of the disease.^2,10,11^ To date, few studies have assessed alterations in metabolomic profiles during the preclinical phase of IBD.^12,13^ Notably, Hua et al. identified crude pathway associations between the biosynthesis of amino acids, nitrogen, primary bile acids, and steroid hormones with future CD risk in two different preclinical cohorts.^13^ However, the associations of these metabolites on other biological factors remain unknown.

Herein, we investigated the serum metabolic changes that occur during the pre-disease phase of CD and assessed whether these metabolomic measures correlated with other biomarkers of CD risk previously identified. This study involved the Crohn’s and Colitis Canada – Genetics, Environment, Microbiome (CCC-GEM) Project, a global prospective cohort, of healthy first-degree relatives (FDRs).

## Materials and methods

### Participant recruitment and study population

The CCC-GEM Project recruited asymptomatic FDRs of CD patients, aged between 6 and 35, from 103 sites worldwide, and prospectively followed them to monitor for the onset of CD. Recruitment occurred between 2008 and 2021, and the current study is based on health record as of July 2021. The cohorts include 5,122 healthy FDR participants, followed by an average of 5.8 years. At recruitment, participants were screened to exclude those with gastrointestinal symptoms or any diagnosis of inflammatory bowel disease, celiac disease, or irritable bowel syndrome. Demographic data were recorded (Supplementary Notes 1, 2) as previously described.^3^ All participants, and/or their guardians, provided written informed consent to participate in the study. The Mount Sinai Hospital Research Ethics Board (Toronto-Managing Center) and all local recruitment centers approved the study (refer to the list in the CCC-GEM Project Research Consortium section).

Participants were contacted every six months to check for any potential new diagnoses of CD. If a participant reported a CD diagnosis, this was confirmed by the treating physician based on clinical, endoscopic, radiographic, and/or histological reports (Supplementary Note 3). A nested case-control cohort was identified to include individuals who developed CD (pre-CD subjects) along with up to four healthy matched control who remained disease-free during the observation period. Matching criteria included age, sex assigned at birth, geographic location, and time of recruitment. This design allowed for detailed phenotypic characterization, including assessments of serum metabolomics, proteomics, gut barrier function assessment (via lactulose-to-mannitol ratio, LMR), fecal calprotectin (FCP), C-reactive protein (CRP), Antimicrobial Antibodies Sum (AS), and gut microbiota analysis (See methods, fig. S1–6). The supplementary methods describe details about the collection of blood, urine, and stool samples at recruitment.

### Assessment of gut barrier function, fecal calprotectin, C-reactive protein, serum proteomics, anti-microbial antibodies, gut microbiota and metabolomics

Assessment of gut barrier function was performed using the urinary fractional excretion of LMR from samples collected at recruitment as previously described.^14^ FCP, a stool biomarker that represents inflammation in the gut, was measured by enzyme-linked immunosorbent assay (ELISA) as previously described.^14^ Assessment of CRP level, serum biomarker that reflects systemic inflammation, was measured by ELISA as previously described.^4^ Proteomic profile of serum was assessed using a proximity extension assay and then quantified by quantitative PCR (Olink Proteomics, Uppsala, Sweden) as previously described.^15^ Anti-microbial antibodies were measured by ELISA, and AS among 6 anti-microbial antibodies was used to reflect the immune response to multiple commensal antigens, as previously described.^4^ Stool microbiota was characterized by 16S ribosomal RNA (16S rRNA) as previously described.^12^ Serum metabolomic measurements were performed with Metabolon using the Metabolon’s DiscoveryHD4™ Platform following manufacturer instructions. Briefly, the platform identified 1026 metabolites with less than 50% of missing values were included in the analysis. The missing data as defined by Metabolon™ were imputed using the minimum values measured for each metabolite by Metabolon inc. (Durham, NC, USA) (For detailed information see Supplementary material).

### Statistical analysis of metabolites associated with risk of Crohn’s disease onset

We used conditional logistic regression, implemented via the *clogit* function in the *survival* package (v 3.5.5), to identify individual CD risk-associated metabolites. *p-*values were adjusted for false discovery rate using the Benjamini-Hochberg method, with an adjusted *p-*value < 0.05 considered statistically significant (see Supplementary Material for details). A subgroup analysis was performed to examine whether the association of the CD risk associated metabolites were consistent across different follow-up durations by dividing the cohort into two sub-cohorts based on the median follow-up time of the nested groups. Conditional logistic regression models were performed on each sub-cohort separately, to assess the association of metabolites with CD onset, with a one-sided *p*-value < 0.05 being considered significant in this analysis. We also conducted a sensitivity analysis to establish whether the metabolites associated with risk of CD onset were independent of other known pre-CD biomarkers. Thus, the association of pre-CD-associated metabolites were re-assessed in the conditional logistic regression model and adjusted for the potential confounding effects by including the continuous variables CRP, FCP, LMR, AS, and microbial alpha diversity (Shannon index after rarefaction at 10,000 reads per sample) (See supplementary methods).

### Statistical analysis of correlation between pre- Crohn’s disease associated metabolites with other markers of Crohn’s disease

To assess the potential pathway(s) leading to CD onset, the pre-CD-associated metabolites were tested for their correlation with 20 stool microbial genera,^12^ alpha diversity, 23 serum proteins,^15^ AS,^4^ CRP,^4,16^ LMR,^14^ and FCP,^14^ biomarkers previously shown to be associated with risk of developing CD. We performed partial Spearman correlation analysis on samples overlapping with serum metabolites and the specified biomarkers (see supplementary materials, Table S1), and adjusted the *p*-values for false discovery rate using the Benjamini-Hochberg method.

To assess whether serum metabolite levels reflect intestinal metabolic activity, we analyzed matched serum and fecal metabolomics data from a subset of individuals who had both biospecimens collected at baseline. We first identified pre-CD-associated serum metabolites that overlapped with fecal metabolites based on PubChem compound identifiers (CHEM_IDs). For each overlapping metabolite, Spearman correlation coefficients were calculated using paired serum and fecal measurements from the same individuals. Resulting *p*-values were adjusted for multiple comparisons using the Benjamini-Hochberg method.

### Mediation analysis between gut microbiota, metabolites, and Crohn’s disease risk

As an exploratory analysis, to further elucidate potential causal pathways among gut microbiota, serum metabolites, and CD development, we performed causal mediation analyses using the R package *mediation* (v4.5.1). Our approach comprised three phases:

1. Variable Selection: *Ruminococcus torques* was selected as the microbial of interest, as it was previously identified as a top contributor to CD risk.^12^ We focused on its three most positively correlated serum metabolites (quinolinate, phenol sulfate, aspartate), selected based on both effect size and statistical significance in our dataset.
2. Bidirectional Modeling: For each metabolite-microbe pair, we conducted separate analyses for both causal directions to explore the directionality of effect between the pairs:
  - Metabolites influencing CD through the *Ruminococcus torques* abundance:
    Mediator model: Linear regression of *Ruminococcus torques* relative abundance on metabolite levels
  - *Ruminococcus torques* abundance influencing CD through the metabolites:
    Mediator model: Linear regression of metabolite levels on *Ruminococcus torques* relative abundance
  - Both mediation paths shared the same logistic regression outcome model, with age, sex, and country included as covariates.
3. Effect Estimation: We computed the Average Causal Mediation Effect (ACME) through 1,000 bias-corrected bootstrap simulation. For each mediation model, we also estimated the Average Direct Effect (ADE), which reflects the effect of the exposure on the outcome not through the mediator. The proportion mediated was calculated as ACME/(ACME + ADE), and its corresponding *p*-value, hereafter referred to as *P*_*medi*_, which represents the significance of the indirect (mediation) effect.
4. The following assumptions were required for the estimates of direct and indirect effects in the mediation analysis to be interpreted appropriately: no unmeasured confounding of the effect of (1) exposure on the outcome; (2) mediator-outcome relationship; (3) exposure-mediator relationship; and (4) mediator-outcome confounder that is itself affected by the exposure.

### Machine learning-based identification of predictive metabolomic signatures for Crohn’s disease

To identify serum metabolomic signatures predictive of CD, we developed a machine learning pipeline based on group-stratified nested cross-validation (using `*GroupKFold*` from scikit-learn) to preserve biological independence between samples. A total of 1,006 quantified serum metabolites which have inter-correlations confidence < 95% were analyzed using an iterative feature selection and evaluation framework. In the first stage, features in each training set were ranked by univariate significance using the ANOVA F-test (`*f_classif*` from scikit-learn). Subsequently, top-ranked features were incrementally grouped into sets of increasing size (2, 4, 8, 16, …, up to 1,024), and each subset was evaluated using a Random Forest classifier (1,000 trees, maximum depth = 8, ‘*RandomForestClassifier*’ from scikit-learn) to determine its predictive performance. Model performance was assessed using the mean area under the receiver operating characteristic curve (AUC-ROC) across five outer folds of cross-validation, with the inner loop used for feature selection and hyperparameter consistency.

Feature importance was derived from the average Gini impurity reduction across trees, and the selection frequency of each feature was recorded to assess robustness across folds. The top 20 features were further visualized based on their average importance and cross-validation stability. All analyses were implemented in Python using scikit-learn (v1.7.0).

## Results

### Characteristics of the nested case-control cohort

The CCC-GEM nested case control cohort (*n* = 389) included 78 subjects who later developed CD (refer as pre-CD), matched with up to four subjects (*n* = 311) who remained healthy. Baseline demographics of this nested cohort are provided in Table 1. Briefly, the median follow-up time was 2.61 years ([Interquartile range, IQR], 1.08–4.51), and the median age at enrollment was 15.0 ([IQR], 11.0–22.0); 56.8% were female. The geographic recruitment was as follows: 84.8% from Canada, 11.8% from Israel, and 3.3% from the United States.

**Table 1.** Characteristics of the nested case-control cohort.

		Remained healthy FDRs (*N* = 311)	Pre-CD FDRs (*N* = 78)	*P*-value
Age at recruitment (years)	Median	16.00	15.00	0.869^c^
	IQR	[11.00, 22.00]	[12.00, 21.00]	
Sex assigned at birth - no. (%)	Female	176 (56.6)	45 (57.7)	0.962^a^
	Male	135 (43.4)	33 (42.3)	
Country - no. (%)	Canada	266 (85.5)	64 (82.1)	0.265^b^
	United States	8 (2.6)	5 (6.4)	
	Israel	37 (11.9)	9 (11.5)	
Relation proband – no. (%)	Sibling	209 (67.2)	62 (79.5)	0.049^a^
	Offspring	102 (32.8)	16 (20.5)	
CD-Multiplex family ( > 1 FDR with CD) – no. (%)	Yes	21 (6.8)	16 (20.5)	4.865 × 10^−4b^
	No	290 (93.2)	62 (79.5)	
Follow-up duration (years)	Median	2.67	2.50	0.632^c^
	IQR	[1.06, 4.42]	[1.30, 4.69]	

Breakdown of the cohort by age, sex assigned at birth, recruitment country, family history (relation proband, CD-multiplex family) and follow-up duration (years). Categorical variables are presented as numbers and percentages; continuous variables are presented as median and IQR. Comparison of categorical variables was performed using the aχ2 test or bFisher’s exact test where appropriate. For continuous variables, cKruskal-Wallis test was applied for median comparisons. CD: Crohn’s disease, FDRs: First-degree relatives, IQR: Interquartile range.

### Baseline metabolomics profiles are significantly associated with the future development of Crohn’s disease

**Figure 1.**
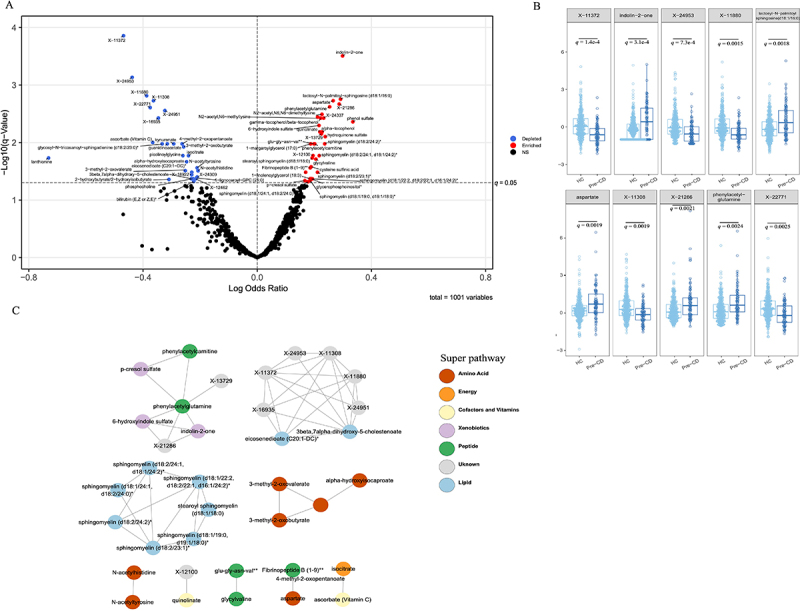
Metabolites associated with future development of CD (A)Volcano plot showing each metabolite’s association with future CD development. The x-axis represents the log of or for CD, and the y-axis shows the log10 of the *q*-value (log transformation for visualization). Conditional logistic regression, adjusted for CD-multiplex family and relation to proband, was used. Red dots represent metabolites significantly upregulated in the pre-CD (*q*-value < 0.05 and OR > 1); and blue dots represent metabolites significantly downregulated (*q*-value < 0.05 and OR < 1). Metabolites abundance values were autoscaling-transformed for comparability(mean-centered, unit-variance scaled), expressed in SD units relative to the cohort mean for each metabolite. (B) A boxplot showing the distribution of the 10 most significant metabolites in the pre-CD and HC groups, based on the regression analysis in (A). Light blue dots denote pre-CD individuals, and dark blue dots represent HC individuals. The *q*-value for each metabolite (same as in A) is displayed above the corresponding pair of boxplots. The Y-axis represents autoscaling-transformed metabolite abundance. (C) Among 63 pre-CD-associated metabolites, 36 had a correlation coefficient above |0.6|, forming nine internal clusters. Partial Spearman’s rank correlation account for the matching factors was used to determine intra-cluster correlation (see methods). Metabolites in these clusters are colored by their super pathway as defined by Metabolon, Inc. Lines between metabolites indicate a correlation coefficient > |0.6|. Feature names include known metabolites and unknowns labeled with “X”- identifiers. ** indicates a compound putatively identified by Metabolon Inc. through matching to its proprietary spectral libraries and orthogonal analytical evidence without a chemical standard; * indicates a compound putatively characterized to a chemical class based on spectral similarity using Metabolon Inc.’s annotation protocols, pending confirmation by a standard. CD: Crohn’s disease, HC: match control; OR: odds ratio, X indicates unknown metabolites; SD: standard deviation.

The metabolomic analysis generated a total of 1026 metabolites from 389 individuals. After dimensionality reduction using principal component analysis (PCA), we observed significant global differences in the metabolomic profiles between healthy matched controls and individuals who later developed CD (fig. S7–11; Supplementary results). Using conditional logistic regression model, we identified 63 metabolites significantly associated with the future onset of CD (Figure 1(A), fig. S12, table S2–4, see methods). Of the 63 metabolites, 34 (54%) were increased in the pre-CD group, while 29 (46%) were decreased compared to healthy matched controls. Figure 1(B) visualizes the distribution of the top 10 most significant metabolites. These metabolites span across several super pathways including Amino Acid, Cofactors and Vitamins, Energy, Lipid, Peptide and Xenobiotics. Among these 63 pre-CD metabolites, 32 showed internal correlations and combining into nine independent clusters (see Figure 1(C)).

A subgroup analysis was conducted to determine whether the identified risk metabolites were influenced by the follow-up time between recruitment and development of CD. For the subgroup of the cohort with a follow-up time shorter than the median follow-up time of the nested case-control cohort (*n* = 190, 1.27 [0.23–2.17] years), 58 out of 63 pre-CD metabolites (92%) remained significantly associated with future risk of CD development (1.05 × 10^−5^ < one-tailed *p*-value < 0.04, table S5). In the subgroup with the follow-up time longer than the median follow-up time of the nested case-control cohort (*n* = 199, 4.68 [2.46–9.56] years), 46 (73%) of the pre-CD metabolites remained significant (6.74 × 10^−5^ < one-tailed *p*-value < 0.05, table S6). These findings indicate that the majority of these metabolic changes are consistent regardless of different time to the onset of CD.

**Figure 2.**
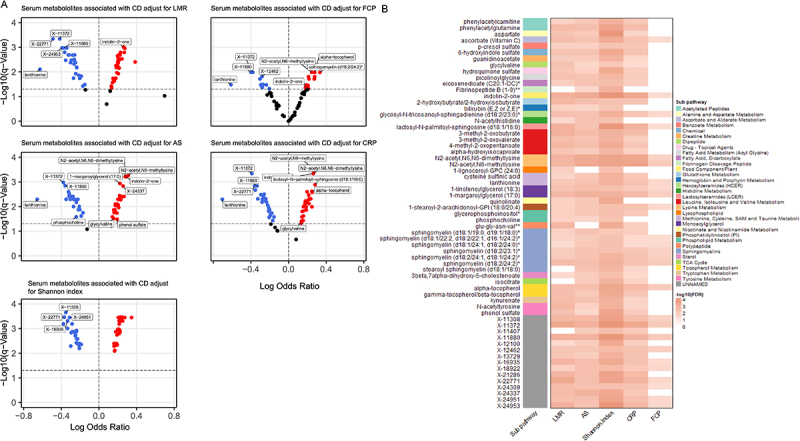
Assessment of potential confounding effect of pre-disease biomarkers on metabolites associated with the risk of developing CD. (A) Volcano plots showing the Fold change of each CD associated metabolite after adjustment for LMR or FCP or as or CRP, or Shannon index covariates separately. Red dots represent significantly increased metabolites in pre-CD group (*q*-value < 0.05, OR > 1), blue dots represent significantly decreased metabolites in the pre-CD group (*q-*value < 0.05, OR < 1). Black dots represent metabolites not significant after covariate adjustment. (B) heatmap of the -log10(*q*-value) of the pre-CD metabolites adjusting for the covariates listed in (A) individually. The left y-axis of the heatmap displays the names of the CD-associated metabolites, with sub-pathways indicated by colors on the right. The x-axis lists the covariates included in the conditional logistic regression. The red intensity of the heatmap squares is proportional to the -log10 of the *q-*value. A darker red tone indicates higher significance, while a white square indicates non-significant associations (*q-*value > 0.05). Feature names include known metabolites and unknowns labeled with “X”- identifiers. ** indicates a compound putatively identified by Metabolon Inc. through matching to its proprietary spectral libraries and orthogonal analytical evidence without a chemical standard; * indicates a compound putatively characterized to a chemical class based on spectral similarity using Metabolon Inc.’s annotation protocols, pending confirmation by a standard. CD: Crohn’s disease; X- indicates unknown but defined compounds; OR: odd ratio. FCP: fecal calprotectin; LMR: lactulose-to-mannitol ratio; CRP: C-reactive protein; AS: serum antimicrobial antibody sum.

Finally, we found that 26/63 (41.3%) of the pre-CD associated metabolites were independent of potential confounding by other pre-CD risk factors (i.e., FCP, LMR, CRP, AS, Shannon Index (individually). In contrast, the other pre-CD associated metabolites lost significance after similar adjustments for these CD risk factors (Figure 2).

### Correlation analysis between pre- Crohn’s disease associated metabolites and other known biomarkers reveal distinct pathways of Crohn’s disease pathogenesis

**Figure 3.**
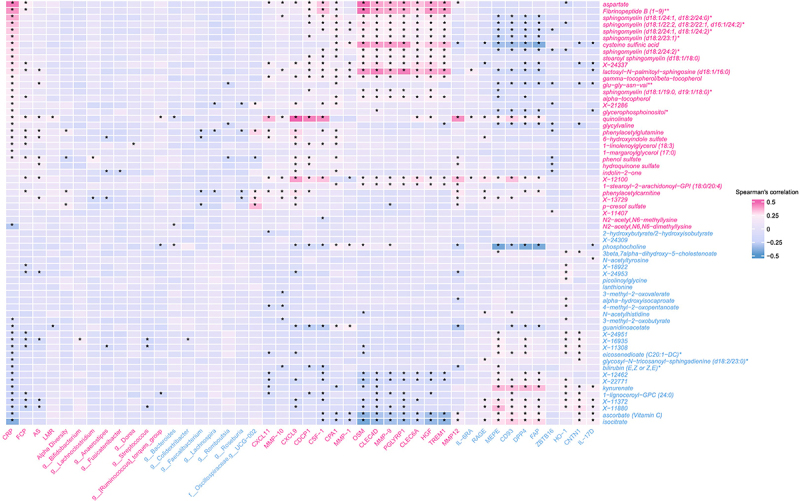
Heatmap of Spearman correlation coefficient between serum proteins, microbiome and clinical variables and CD associated metabolites. Partial Spearman’s rank correlation accounts for the matching conditions (age, sex assigned at birth, country, and follow-up duration), CD-multiplex family, and relation to proband (sibling vs offspring). Only correlations with *q*-value < 0.05 are represented by a star*; the Y-axis represents the 63 pre-CD associated metabolites. The x-axis represents previous biomarkers of CD. Blue labeling indicates biomarkers associated with decreased risk of CD, while red labels indicate biomarkers associated with increased risk of CD (see methods). The color intensity indicates the coefficient of correlation from red (indicating positive correlation) to blue (indicating negative correlation). Feature names include known metabolites and unknowns labeled with “X”- identifiers. ** indicates a compound putatively identified by Metabolon Inc. through matching to its proprietary spectral libraries and orthogonal analytical evidence without a chemical standard; * indicates a compound putatively characterized to a chemical class based on spectral similarity using Metabolon Inc.’s annotation protocols, pending confirmation by a standard. CD: Crohn’s disease; FCP: fecal calprotectin; LMR: lactulose-to-mannitol ratio; CRP: C-reactive protein; AS: serum antimicrobial antibody sum.

We performed a correlation analysis of pre-CD metabolites with different biomarkers that we previously identified as risk factors for CD onset (See methods). CRP showed the highest proportion of significant correlations (41/63 metabolites, 65.1%), followed by serum proteins associated with CD risk (range: 3–29/63, 4.7–46.0%), FCP (20/63, 31.7%), AS (13/63, 20.6%), microbiome alpha diversity (4/63, 6.3%), microbial taxa (1–6/63, 1.5–9.5%), and LMR (2/63, 3.1%). Notably, serum proteins exhibited substantial heterogeneity, with proteins like matrix extracellular phosphoglycoprotein (MEPE) correlating with 46.0% of metabolites while Interleukin-6 Receptor Alpha (IL-6RA) correlated with only 4.7%. Overall, we observed distinct correlation patterns where certain metabolites showed associations with multiple biomarkers while others correlated with only one specific biomarker (see Figure 3, table S12–18, fig. S13–20).

#### Role of tryptophan

We found that quinolinate, a tryptophan-related metabolite, was positively correlated with chemokine and cytokine proteins, including C-X-C motif chemokine (CXCL) 9 (ρ = 0.51, *q*-value = 5.92 × 10^−21^), CXCL11, and matrix metalloproteinases (MMP12 and MMP10) in addition to other pre-CD biomarkers including CRP, FCP, AS, LMR, and with increased relative abundance of *Ruminococcus torques*. Additionally, we also found that kynurenate, a derivate of tryptophan metabolism associated with a decreased risk of CD, was positively correlated with proteins protective against CD including MEPE, cluster of differentiation 93 (CD93), dipeptidyl peptidase-4 (DPP4), and fibroblast activation protein (FAP). Kynurenate was also inversely correlated with CRP (table S12–18).

#### Role of vitamin C

We found that ascorbate (vitamin C) was positively correlated with six out of 10 proteins associated with decreased risk of CD, while negatively correlated with 10 out of 15 proteins associated with increased risk of CD. While ascorbate was negatively correlated with CRP, it was not correlated with FCP and any pre-CD associated microbial taxa (table S12–18).

#### Role of fibrinopeptide B

We found that aspartate and Fibrinopeptide B (1 − 9) were both positively correlated with 14 and 10 out of 15 proteins, respectively, that were associated with increased risk of CD but had limited correlation with proteins associated with decreased risk of CD onset. In addition, aspartate demonstrated the strongest positive correlation with CRP (ρ = 0.457, *q*-value = 2 × 10^−19^) and with FCP (ρ = 0.246, *q*-value = 2 × 10^−4^) (table S12–18).

#### Association of metabolomics with microbiota

The genera *Anaerostipes, Faecalibacterium, Romboutsia, Lachnoclostridium*, and *UCG-002* (family Oscillospiraceae) exhibited significant correlations with multiple metabolites (Table S17). Metabolites known to be microbiome-metabolized – particularly p-cresol sulfate and inulin derivatives – showed strong directional associations: *Faecalibacterium* was negatively correlated with p-cresol sulfate (ρ = −0.173, *q*-value = 0.03), *phenylacetylcarnitine* (ρ = −0.216, *q*-value = 0.002) and *phenylacetylglutamine* (ρ = −0.202, *q*-value = 0.008); Indole-2-one demonstrated negative correlations with both *Anaerostipes* (ρ = −0.223, *q*-value = 0.002) and *Fusicatenibacter* (ρ = −0.187, *q*-value = 0.020). Interestingly, Oscillospiraceae *UCG-002*, was positively correlated with pre-CD metabolites including p-cresol sulfate (ρ = 0.345, *q*-value = 7 × 10^−9^), phenylacetylglutamine (ρ = 0.295, *q*-value = 4 × 10^−6^) and phenylacetylcarnitine (ρ = 0.260, *q*-value = 1 × 10^−4^). Microbial alpha diversity was positively correlated withp-cresol sulfate (ρ = 0.19, *q*-value = 0.009 and phenylacetylglutamine (ρ = 0.18, *q*-value = 0.009) but negatively correlated with phenol sulfate (ρ = −0.18, *q*-value = 0.009), and phenylacetylcarnitine (ρ = −0.16, *q*-value = 0.02). We also found that increased phosphocholine was negatively correlated with the relative abundance of *Ruminococcus Torques* (ρ = −0.176, *q*-value = 0.029).

Hydroquinone sulfate (ρ = 0.165, *q*-value = 0.024), lactosyl-N-palmitoyl-sphingosine (ρ = 0.153, *q*-value = 0.024), and phenol sulfate (ρ = 0.160, *q*-value = 0.024) were positively correlated with microbial antibodies. Quinolinate was positively correlated with microbial antibodies (ρ = 0.156, *q*-value = 0.024) and gut permeability (ρ = 0.217, *q*-value = 0.002).

#### Role of the sphingolipids

We found that multiple pre-CD associated metabolites belong to the sphingolipids class. For example, sphingomyelins (d18:1/24:1, d18:2/24:0) were positively correlated with CRP (ρ = 0.367, *q*-value = 1.84 × 10^−12^) but not with FCP. In addition, many risk associated sphingolipids were positively correlated with CD risk associated proteins including Oncostatin M (OSM), C-Type Lectin Domain Family 4 Member D (CLEC4D), MMP9, Peptidoglycan Recognition Protein 1 (PGLYRP1), Hepatocyte Growth Factor-Regulated Tyrosine Kinase Substrate (HGS) and Triggering Receptor Expressed on Myeloid cells 1(TREM1). Conversely, these sphingolipids were negatively correlated with proteins associated with decreased CD risk such as MEPE, CD93, DPP4, FAP (table S12, 13 and 18).

### Correlation between serum and fecal levels of pre- Crohn’s disease metabolites

Among the 63 serum metabolites associated with future CD risk, 26 (41.3%) were also detected in matched fecal metabolomics profiles from the same individuals (*n* = 122). We evaluated the correlation between serum and fecal concentrations for these overlapping metabolites. Most metabolites showed non-significant correlations across compartments; however, three metabolites exhibited statistically significant correlations after FDR correction: phenol sulfate (ρ = 0.31, *q*-value = 0.010), indolin-2-one (ρ = 0.25, *q*-value = 0.044), and lactosyl-N-palmitoyl-sphingosine (ρ = 0.25, *q*-value = 0.044) (see Table S19).

### Bidirectional mediation analysis of ruminococcus torques and its correlated metabolites in Crohn’s disease risk

**Figure 4.**
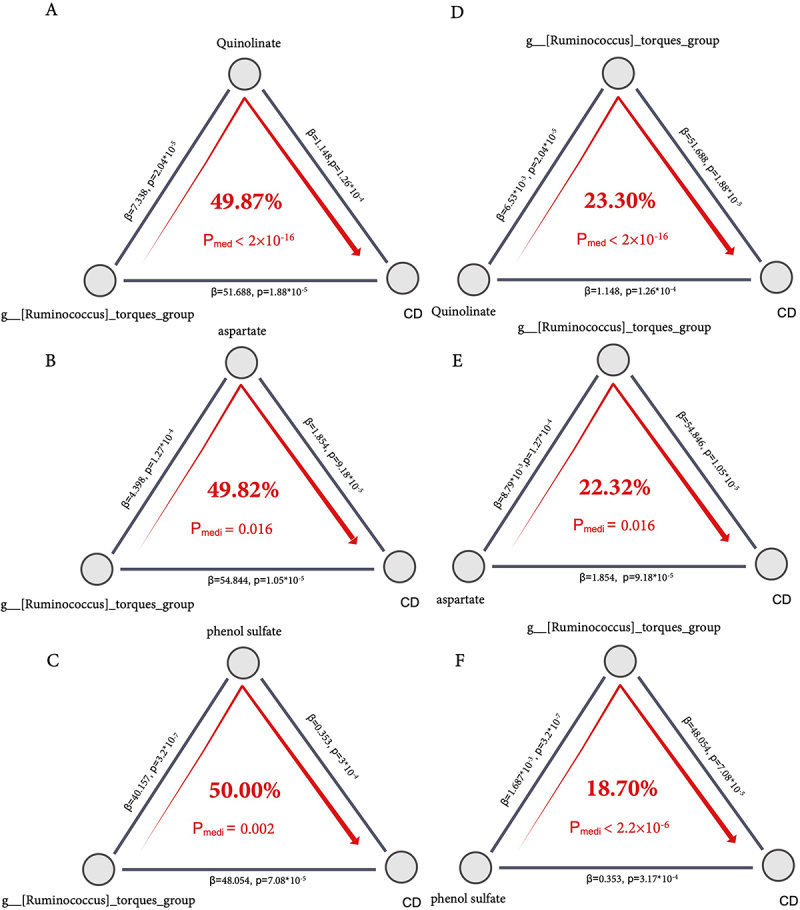
Bidirectional mediation analysis of *Ruminococcus torques* and its associated metabolites in CD risk. Each panel illustrates one direction of the mediation model: (A – C) *Ruminococcus torques* relative abundance influencing CD through the metabolites, and (D – F) metabolites influencing CD through the relative abundance of *Ruminococcus torques*. The numerical values along the borders of the triangle represent the effect sizes (β) from the mediator or outcome models, along with their corresponding P-values. The percentage inside the triangle indicates the proportion mediated, calculated as ACME/(ACME + ADE). *P*_*med*_ represents the statistical significance of the mediation (i.e., indirect) effect estimated via 1,000 bias-corrected bootstrap simulations. All models were adjusted for age, sex, and country. CD, Crohn’s disease; ACME, average causal mediation effect; ADE, average direct effect; β, regression coefficient; *P*_*medi*_, P-value for the mediation effect.

To explore the potential mediating roles of *Ruminococcus torques* and its correlated metabolites, we performed bidirectional causal mediation analyses using the three most positively correlated serum metabolites – quinolinate, phenol sulfate, and aspartate – selected based on both effect size (Spearman’s ρ > 0.11) and statistical significance (*p*-value < 0.02) in our dataset, as both exposures and mediators (Figure 4).

When modeling the microbe (*Ruminococcus torques*) as the exposure and metabolites as mediators, we observed that phenol sulfate mediated 50.00% of the total effect on CD risk (*P*_*medi*_ < 0.002), followed by quinolinate (49.87%, *P*_*medi*_ = 2.00 × 10^− 1 6^), and aspartate (49.82%, *P*_*medi*_ = 0.016). These results suggest that a substantial proportion of the microbe’s association with CD may be mediated via altered metabolite levels.

In the reverse direction, with each metabolite as the exposure and *Ruminococcus torques* relative abundance as the mediator, the mediation effects remained significant, albeit lower in magnitude. Specifically, *Ruminococcus torques* showed 23.30% mediation (*P*_*medi*_ < 2.2 × 10^−^1^6^) for quinolinate, 22.32% for aspartate (*P*_*medi*_ = 0.016) and 18.7% for phenol sulfate (*P*_*medi*_ < 2 × 10^−6^). Collectively, these findings support the possibility of a bidirectional association between *Ruminococcus torques* and a subset of serum metabolites that were statistically correlated with its abundance in our dataset.

### Machine learning identifies robust metabolomic signatures for Crohn’s disease prediction

**Figure 5.**
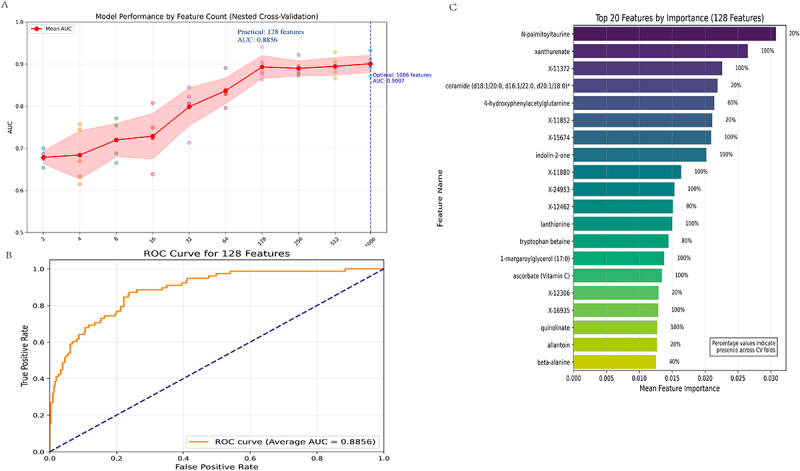
Performance of the metabolite-based Random Forest model for CD prediction using nested cross-validation. (A) model performance measured by AUC across varying numbers of features, evaluated using nested CV. The red line shows the mean AUC, with the shaded area representing standard deviation across five folds. The vertical dashed blue line marks the optimal model with 1006 features (AUC = 0.9007), while a more practical model using 128 features (AUC = 0.8856) is also highlighted. Each dot represents the AUC result from one of the five cross-validation folds. The red line indicates the mean AUC, and the shaded area represents the 95% confidence interval (CI). (B) ROC curve for the model using 128 selected features, yielding an average AUC of 0.8856 across five CV folds. The dashed line represents the mean performance under random classification (AUC = 0.5). (C) top 20 most important features (out of the 128 selected), ranked by mean feature importance across five CV folds. Bar lengths indicate mean importance, and the percentages on the right represent the proportion of folds in which each feature was selected (e.g., 100% = selected in all five folds; 20% = selected in one Fold). Feature names include known metabolites and unknowns labeled with “X”- identifiers. CD, Crohn’s disease; AUC, area under the curve; ROC, receiver operating characteristic.

Using a Random Forest classifier with group-stratified cross-validation, we systematically evaluated the predictive performance of serum metabolites across varying feature set sizes (Figure 5(A)). Model discrimination improved with increasing numbers of features, reaching optimal performance with the full set of 1006 metabolites (mean AUC = 0.9007). Importantly, a compact panel of the top 128 ranked features retained near-maximal performance (mean AUC = 0.8856). The ROC curve of the 128-feature model demonstrated high sensitivity and specificity between pre-CD cases and healthy controls (Figure 5(B)).

To further investigate the biological relevance and robustness of these features, we examined the top 20 contributors ranked by mean feature importance across cross-validation folds (Figure 5(C)). Several metabolites previously identified in our regression analyses as associated with CD risk – such as X-11372, X-24953, indolin-2-one, X-11880, X-12462, lanthionine, 1-margaroylglycerol (17:0), ascorbate (vitamin C), X-16935, and quinolinate – were among the top 20 important features. Notably, these markers were also consistently selected across all cross-validation fold, reinforcing their stability and reliability as predictive biomarkers of CD onset.

## Discussion

Previous studies aimed at understanding the pathogenesis of CD suggest that it is a multifactorial condition. Thus, a multi-omic approach is necessary to capture the various factors involved in its development. Our study focused on serum metabolomics while also taking advantage of the extensive phenotypic dataset from the CCC-GEM project, which includes other biomarkers linked to CD risk. Serum metabolomics is particularly valuable because it provides insights into metabolites absorbed by and interacting with the host or its microbiome. Moreover, metabolomic analysis captures chemical substrates, intermediates, or end products of enzyme-mediated reactions, allowing for a comprehensive understanding of the host microbiome interaction that may lead to CD onset.

We found evidence that a total of 63 serum metabolites were differentially abundant in individuals that later develop CD compared to those that remain healthy. Importantly, 26 of these metabolites, including ascorbate, specific sphingolipids, and lysine derivatives, maintained significant associations after adjustment for established risk factors including FCP, suggesting they may represent primary drivers of pathogenic pathways rather than merely reflecting subclinical inflammation. Subgroup analysis further revealed that the majority of these metabolites remained significantly altered even in individuals with longer follow-up times, supporting that these metabolic changes preceded CD onset. Moreover, most of the pre-CD metabolites were correlated with other biomarkers of CD risk, supporting their relevance in disease pathogenesis. Interestingly, a large portion of pre-CD metabolites were correlated with proteomic markers associated with CD while some pre-CD metabolites were defined by different patterns of correlations that may reflect different pathways of pathogenesis.

Previous studies have identified proteins associated with CD risk.^15,16^ However, it remains unknown how these relate with specific metabolites. The addition of proteomic marker of CD along with metabolome could help to understand biological interactions that could lead to CD onset.^17^ For example, we found that quinolinate had a particularly strong correlation with CXCL9 and to a lesser extent AS, FCP and CRP. This is particularly interesting given that CXCL9 was previously associated with CD risk,^15^ is known to drive systemic inflammation,^18^ and is highly expressed in intestinal tissues of CD patients.^19^ We found that quinolinate was also positively correlated with *Ruminococus torques abundance*, which was previously described as being a major CD risk associated taxa.^12^ In addition, bacteria can synthesize quinolinic acid from aspartate and glyceraldehydes-3-phosphate via the aspartate pathway.^20,21^ In our study we found that the aspartate level was also associated with CD risk and positively correlated with CXCL9. Interestingly, some studies found that aspartate facilitates IL-1β production in inflammatory macrophages^22^ while other studies found that microbiota-derived aspartate drives pathogenic Enterobacteriaceae expansion in the inflamed gut.^23^ This potentially suggesting that the microbiome can be contributing to higher abundance of CD-associated metabolites (like aspartate and its derivative quinolinate) in the gut, which are subsequently absorbed and transformed by the host. Based on our exploratory mediation analysis, we found evidence of a potential bidirectional relationship between *Ruminococcus torques* and several serum metabolites associated with CD. However, further mechanistic studies are needed to prove this bidirectional relationship. Overall, our findings reveal novel associations linking the gut microbiota to elevated levels of aspartate and its derivative quinolinate, two metabolites associated with increased CD risk, suggesting a potential microbial contribution to their abundance. In support of this, we also observed that a subset of serum metabolites – including phenol sulfate and indolin-2-one – showed significant correlations with their fecal levels, further suggesting a microbial or intestinal origin.

Our study also demonstrated that p−cresol sulfate was associated with increased risk of CD onset. Interestingly p-cresol sulfate was negatively correlated with the relative abundance of *Faecalibacterium*, a genus often reported as being protective of CD risk. Indeed, p-cresol sulfate is a product of tyrosine fermentation, mostly performed by bacteria including taxa such as Coriobacteriaceae or *Clostridium*,^24^ followed by sulfation by the host’s cells as part of a detoxification mechanism.^25^ It was shown that p-cresol is produced by fermentation of dietary tyrosine and phenylalanine^24^ and that increased dietary protein intake raised the amount of p-cresol recovered in the feces and urine. This suggests that increased level of p-cresol might be related to a diet rich in protein,^26^ which may also contribute to risk of CD. However, a meta-analysis found that among the dietary sources of protein, the risk of IBD increased only with increased amount of total meat intake, while the consumption of protein from dairy products was found to be protective against the IBD risk.^27^ Although we found no association between p-cresol sulfate with either gut permeability, CRP, host proteomic or FCP, a previous study described that p-cresol sulfate in excess was shown to be genotoxic for colonocytes^28^ and induced neutrophil oxidative burst activity in vitro.^29^

Interestingly, we also found that increased ascorbate (Vitamin C) and isocitrate were associated with a decreased risk of CD. Both metabolites showed evidence of a negative correlation with CRP, as well as with several proteins associated with increased CD risk, including CSF-1, CPA-1, MMP-1, OSM, CLEC4D, MMP-9, PGLYRP-1, CLEC6A, HGF, TREM1 and MMP12. Ascorbate is well known for its antioxidant properties in humans, particularly in scavenging reactive oxygen species.^30^ It is also involved in all phases of wound healing^31^ and supports immune defense by enhancing various cellular functions of both the innate and adaptive immune systems.^32^ Individuals with vitamin C deficiency have been reported to be more prone to infection. Our study found serum vitamin C remained independently associated with a reduced risk of CD after adjusting for CD risk biomarkers including microbiome diversity, AS, inflammation or gut permeability. Since ascorbate is an essential nutrient that humans cannot synthesize, our study suggests that the low level of vitamin C levels might reflect low dietary intake potentially promoting the risk of developing CD. This insight could inform preventative strategies aimed at increasing the abundance of these metabolites to potentially reduce the risk of developing CD. Supplementing healthy individuals with dietary or high-dose vitamin C has been shown to improve neutrophil chemotaxis,^33^ while high-dose vitamin C administration to animal models ameliorated colitis.^34^ Vitamin C deficiency has also been commonly identified in CD patients.^35–37^ Given that vitamin C is abundant in fruits and vegetables,^38^ addressing this deficiency through dietary management could potentially reduce the risk of developing CD.

Beyond the identification of individual metabolite associations with CD risk, we also evaluated their combined predictive potential and confirmed that a panel of 128 serum metabolites discriminated pre-CD and healthy controls. Notably, several CD-associated metabolites previously identified through regression – including quinolinate, indolin-2-one, X-11372, and ascorbate – ranked among the most important and stably selected features across validation folds, reinforcing their relevance as robust predictive biomarkers.^39–45^

This study has multiple strengths including the assessment of a large set of biomarkers of CD allowing for the first comprehensive multi-omics assessment of CD pathogenesis. However, this study also has some limitations. Notably, we could not determine the intricate causal relationship between metabolites and other factors. Additionally, we could not assess the stability of the identified CD risk factors over time. Finally, our findings primarily reflect North American and Israeli populations, which may limit generalizability to other settings.

In conclusion, our study provided strong evidence that serum metabolomics could reflect multiple risk pathways of CD. Thanks to the integration of multiple datasets, we found additional evidence of the role of protein involved in immune response, in addition to the critical role of the microbiome in promoting the risk for CD. Thus, the identification of novel metabolite pathways that contribute to CD development offers potential opportunities for early detection of individuals at risk of CD and points to potential strategies for intervention to prevent the onset of disease. Future studies employing animal colitis models – combined with longitudinal sampling, functional metagenomics, dietary assessments, and targeted manipulations in model systems – are essential to establish causal links between identified metabolites (e.g., p-cresol sulfate) and CD inflammatory pathways leading to CD onset.

## Supplementary Material

Supplemental Material

Supplemental Material

Supplementary_Note_2.docx

Supplementary_Note_1.docx

Supplementary_Note_3.docx

## Data Availability

Requests for raw and analyzed data should follow the instructions given at http://www.gemproject.ca/data-access/. The raw microbiome data are publicly accessible under Accession: PRJNA685746. All submissions will be reviewed by the GEM Project Operating Committee to ensure that the requested samples/data will not interfere in any way with the intended GEM Project analysis of the nested cohort as per the original GEM Project Study Design and is not a duplication of analysis already ongoing. Those proposals meeting this evaluation will be distributed to all members of the GEM Project Steering Committee (GPSC) for review and open discussion. This review will focus on the global scientific merit of the proposal. This review will assess the basic scientific merit and the availability of requested samples and data, ensuring there is no compromise of the original intent of the GEM project. It would be of value to contact a member of the Steering Committee who could help sponsor your application. Those projects achieving majority vote of approval at the GPSC will be informed that the GEM Project will provide a letter of support stating that the requested samples or data will be made available to the applicants once the applicant receives funding from a granting agency that applies an independent peer review process to the proposal. The criteria to be used for review of all submissions will include the “scientific relevance” of the proposal and the judged availability of biological material requested. The budget to be requested from a funding agency must allow for any expenses in processing samples or in setting up the appropriate queries of the database. The intent is to allow sufficient time for applicants to consider submission for funding opportunities. The code that supports the finding of this study are available for public download at https://github.com/MingyueXue-luna/Serum-metabolites-and-CD.

## Abbreviations:

CD: Crohn’s Disease;
IBD: Inflammatory Bowel Disease;
CCC-GEM: Crohn’s and Colitis Canada – Genetics, Environment, Microbiome;
FDR: First-Degree Relatives;
NCC: nested case-control design;
pre-CD: individuals who later developed Crohn’s disease;
FCP: Fecal Calprotectin;
CRP: C-Reactive Protein;
LMR: Lactulose-to-Mannitol Ratio;
ELISA: enzyme-linked immunosorbent assay;
AS: Antimicrobial Antibodies Sum;
16S Rrna: 16S ribosomal RNA;
PCA,: Principal Component Analysis
AUC: Area Under the Curve;
AUC-ROC: Area Under the Receiver Operating Characteristic Curve; IQR, Interquartile Range;
PERMANOVA: Permutational Multivariate Analysis of Variance;
CXCL: C-X-C motif chemokine ligand;
MMP: matrix metalloproteinases;
MEPE: matrix extracellular phosphoglycoprotein;
DPP4: dipeptidyl peptidase-4;
FAP: fibroblast activation protein;
CD93: cluster of differentiation 93
OSM: Oncostatin M;
CLEC4D: C-Type Lectin Domain Family 4 Member D;
PGLYRP1: Peptidoglycan Recognition Protein 1;
HGS: Hepatocyte Growth Factor-Regulated Tyrosine Kinase Substrate;
TREM1: Triggering Receptor Expressed on Myeloid Cells 1;
s.d: Standard Deviation;
PEA: Proximity Extension Assay;
NPX: Normalized Protein Expression;
LOD: Limit of Detection;
ASV: Amplicon Sequence Variant;
ASCA: Anti-Saccharomyces Cerevisiae Antibodies
Ig: Immunoglobulin;
OmpC: anti-Escherichia coli outer membrane porin C;
UPLC-MS/MS: Ultra Performance Liquid Chromatography-Tandem Mass Spectrometry;
PFPA: Perfluoropentanoic Acid;
FA: Formic Acid;
OR: Odds ratio; HC: healthy control;
ρ (rho: Spearman’s rank correlation coefficient;
*q*-value: False discovery rate (FDR)-adjusted *p-*value;
ACME: Average Causal Mediation Effect;
ADE: Average Direct Effect; β, Regression Coefficient;
*P*_*medi*_: *P-value* for the mediation effect.
