# Supplementary material for: Metabolomics reveal distinct molecular pathways associated with future risk of Crohn’s Disease
Source: Gut Microbes. 2025 Sep 5;17(1):2546998. doi: 10.1080/19490976.2025.2546998 (PMC12416195; doi:10.1080/19490976.2025.2546998)
Supplement: Supplemental Material [file KGMI_A_2546998_SM5325.docx]

**Supplementary Methods**

***Specimen collection***

Serum samples

A serum separating tube was used to collect a blood sample through venipuncture. After waiting for 15-30 minutes, the samples were centrifuged to separate the serum from the cells. The supernatants from the serum separating tubes were then sent to central processing laboratories located at the University of Toronto (for North American sites) and the Shaare Zedek Medical Centre (for Israeli sites) for processing. Once cataloged, the serum samples were frozen in a -80°C freezer with continuous monitoring to enable metabolic, protein, C-reactive protein (CRP), and antimicrobial antibodies (AS) profiling as part of the study.

Stool samples

Participants were provided with a stool commode from Fisher Scientific (Waltham, MA) and a polypropylene specimen collection container from Starplex Scientific Inc. (Etobicoke, ON) to collect stool samples. These samples were immediately frozen in the participants’ home freezers. To maintain their frozen state during transportation to the recruitment center, the samples were packed with ice packs. Upon arrival, they were stored in monitored -80°C freezers. They were periodically batch shipped to the Crohn’s and Colitis Canada – Genetics, Environment, Microbiome (CCC-GEM) Project's core facility using World Courier Cold Chain Logistics, accompanied by temperature probes and dry ice to keep the samples frozen during transport. Once at the core facility, the samples were cataloged and immediately stored in -80°C freezers equipped with emergency backup power and dual temperature monitoring systems.

Urine samples

To measure the fractional excretion of lactulose divided by that of mannitol (lactulose-to-mannitol ratio, LMR), urine samples were collected under specified conditions[39]. Before probe administration, participants refrained from consuming alcohol, aspirin, and other non-steroidal anti-inflammatory agents for at least five days. A standard solution containing lactulose (5 g), mannitol (2 g), sucrose (100 g), and flavored drink crystals (1.5 g) dissolved in 500 mL of tap water was ingested before bedtime. The following morning, the urine was collected into a container containing 5 mL of thymol solution and transported to the study center. At the center, the total urine volume was measured, and 5 mL aliquots were frozen at -80°C.

***Proteomics analysis***

The serum proteomic profile was measured using serum samples via a proximity extension assay (PEA) and quantified by quantitative polymerase chain reaction (qPCR) (Olink® Proteomics, Uppsala, Sweden). Pairs of antibodies with complementing DNA oligonucleotide tails bind to the target protein in proximity of each other, creating a DNA sequence that is elongated by DNA polymerase and then read out by qPCR. This sequence is subsequently amplified by microfluidic qPCR, and normalized protein expression (NPX) data are generated using a delta-delta cycle threshold calculation method[40]. Using this platform, we analyzed proteins from five different panels (Olink® Target 96 panels) involved in inflammation, cardiometabolic, cardiovascular and immune-response processes. NPX values below the limit of the detection (LOD) were imputed using the LOD value. Missing values, reported for measurements that failed to achieve validity in the PEA assay, were imputed using the MICE R package with the Multivariate Imputation by Chained Equations method, as described previously[15].

***Gut microbiota analysis***

As previously described[12], stool DNA was extracted using the QIAamp DNA Stool Mini Kit (Qiagen, Hilden, Germany) with some modifications. This included the addition of 0.7 g of garnet beads (0.8 mm) followed by vortexing for three minutes, then 1 g of glass beads (0.1 mm) and vortexing for eight minutes, and a final five-minute incubation in a 90°C water bath. The V4 hypervariable region of the bacterial 16S ribosomal RNA (16S rRNA) was amplified using the 515F/806R primer pair and sequenced in paired-end mode (2 x 150 base pairs) using the MiSeq platform (Illumina Inc., San Diego, CA, USA). For preprocessing of paired-end reads, QIIME 2™ software (version 2020.6) was employed, which involved denoising with the dada2 plugin, truncation reads based on a sample of 30,000 read pairs, denoising with an error model on 1,000,000 reads, and chimeras detection using the “consensus” method with independent pooling. These steps were performed on a per-batch basis, and post-denoising, the per-batch artifacts were merged for taxonomic assignment. A median of 63,486 sequences per sample was obtained after denoising, merging and chimera filtering. Classification of reads was performed using classify-sklearn with the silva-138-99-nb-classifier for taxonomic assignment of each Amplicon Sequence Variant (ASV). Finally, the resulting ASV table was summarized into taxonomic ranks using the phyloseq package (v1.34.0). The resulting taxonomic table was transformed into relative abundance values, and 415 genera were identified for further analysis.

To define if a taxon is associated with increased or decreased risk of CD, we calculated the spearman rank correlation between each decile of the risk score and the log (median relative abundance) for the individuals that fall within each microbiome risk score decile as described previously[12]. This assessment was performed using the R statistical software (v4.0.3) using the *cor.test* function and the spearman correlation index.

***Fecal calprotectin***

The fecal calprotectin (FCP) concentration was determined using the BÜHLMANN fCAL® ELISA test (Schöonrenbuch, Switzerland), which was carried out according to the manufacturer's instructions. The calprotectin concentration was calculated using the average of duplicates. A working range of 30-1800 μg/g for FCP was selected in accordance with the manufacturer's protocol (as illustrated in the fig. S3). To prepare the sample, 50 to 100 mg of stool was mixed with the extraction buffer and vortexed for 30 minutes, while considering the appropriate dilution factors. The homogenate was centrifuged twice (5 minutes each time), and the supernatant was collected for ELISA. The ELISA was conducted as per the manufacturer's protocol, and the absorbance was measured at 450 nm using a microtiter plate reader.

***C-reactive protein***

CRP level was assessed using a Meso Scale Discovery (MSD, Gaithersburg, MD) assay at Prometheus Laboratory (San Diego, CA) according with the manufacturer's instructions. According to the manufacturer's protocol, the minimum detectable concentration was 0.1 mg/L, so any values below this level were considered to be equal to 0.1 mg/L. The upper limit of detection was 203.8 mg/L, and none of the samples exceeded this limit (fig. S4).

***Assessment of Intestinal Barrier***

Following the urine collection as described above, the concentrations of lactulose and mannitol were measured via high-performance liquid chromatography (HPLC)[3]. The fractional excretion of lactulose and mannitol was calculated for each subject as the ratio of the total amount of each saccharide probe excreted in the urine to the total oral dose of the probe[14]*.* Next, the LMR was calculated by dividing the fractional excretion of lactulose by the fractional excretion of mannitol for each subject (see fig. S5).

***Assessment of serum Anti-microbial Antibodies***

As previously described[4], we measured anti-microbial antibodies additively, including anti-Saccharomyces cerevisiae antibodies (ASCA) immunoglobulin (Ig) A and IgG, anti-Escherichia coli outer membrane porin C (OmpC), and anti-flagellin antibodies (anti- CBir1, anti-Fla2, anti-A4-FlaX) in the serum using a commercialized enzyme-linked immunosorbent assay at Prometheus Laboratories (San Diego, CA)[41]. Reference ranges from healthy individuals were used to determine positivity of each marker[41], and the cumulative number of positive antibodies (AS) was calculated for each sample, ranging from 0-6 (fig. S6).

***Assessment of serum metabolomic***

Serum metabolomic measurements were performed with Metabolon using Ultrahigh Performance Liquid Chromatography-Tandem Mass Spectroscopy (UPLC-MS/MS) as defined by Metabolon’s DiscoveryHD4™ Platform. All methods utilized a Waters ACQUITY ultra-performance liquid chromatography (UPLC) and a Thermo Scientific Q-Exactive high resolution/accurate mass spectrometer interfaced with a heated electrospray ionization (HESI-II) source and Orbitrap mass analyzer, operated at 35,000 mass resolution. The sample extract was dried and then reconstituted in solvents compatible with each of the four methods. Each reconstitution solvent contained a series of standards at fixed concentrations to ensure injection and chromatographic consistency. One aliquot was analyzed using acidic positive ion conditions, chromatographically optimized for more hydrophilic compounds. In this method, the extract was gradient eluted from a C18 column (Waters UPLC BEH C18-2.1x100 mm, 1.7 µm) using water and methanol, containing 0.05% perfluoropentanoic acid (PFPA) and 0.1% formic acid (FA). Another aliquot was analyzed using acidic positive ion conditions; however, it was chromatographically optimized for more hydrophobic compounds. In this method, the extract was gradient eluted from the same aforementioned C18 column using methanol, acetonitrile, water, 0.05% PFPA, and 0.01% FA and was operated at an overall higher organic content. Another aliquot was analyzed using basic negative-ion-optimized conditions with a separate, dedicated C18 column. The basic extracts were gradient eluted from the column using methanol and water but with 6.5 mM Ammonium Bicarbonate at pH 8. The fourth aliquot was analyzed via negative ionization, following elution from a HILIC column (Waters UPLC BEH Amide 2.1x150 mm, 1.7 µm), using a gradient consisting of water and acetonitrile with 10 mM Ammonium Formate at pH 10.8. The MS analysis alternated between MS and data-dependent MSn scans using dynamic exclusion. The scan range covered 70-1000 m/z.

Raw data were extracted, peak-identified, and QC processed using Metabolon’s hardware and software systems. These systems are built on a web service platform utilizing Microsoft .NET technologies, which run on high-performance application servers and fiber-channel storage arrays organized in clusters to provide active failover and load balancing. Compounds were identified by comparison with library entries of purified standards or recurrent unknown entities. For studies spanning multiple days, a batch normalization step was performed to correct variation resulting from instrument inter-day tuning differences. Essentially, each compound was corrected in run-day blocks, by registering the medians to equal one (1.00) and normalizing each data point proportionately (termed the “block correction”). For studies that did not extend a single day of analysis, normalization was not necessary, other than for data visualization purposes. Then, each metabolite in each batch-normalized dataset was group-normalized against the median of the health match control samples.

This comprehensive analysis enabled the identification of 1026 metabolites with less than 50% missing values, including 843 named metabolites and 183 unnamed metabolites. The missing data as defined by Metabolon™ were imputed using the minimum values measured for each metabolite by Metabolon inc. (Durham, NC, USA). Finally, the metabolic data from 389 participants were included in the study, and the 1,026 metabolites were subjected to autoscaling for use in assessing their association with future risk of CD onset (Table S1). This transformation allows for the comparison of relative changes across metabolites with different baseline abundances and variances.

***Statistical Analysis***

Multivariate Analyses of Metabolomic, Proteomic, and Microbiome Profiles

To assess global host molecular patterns associated with CD onset, we applied complementary dimensionality reduction and dissimilarity methods. Principal component analysis (PCA) was performed on the 1,026 host serum metabolomic profiles, proteomic data, and gut microbiota using the *PCAtools* package in R with Euclidean distance[42]. Assessment of differences in the principal components (PCs) of metabolites between the pre-CD group and healthy matched control groups was done using Permutational Multivariate Analysis of Variance (PERMANOVA) with the 'adonis' function from the vegan package in R (a total of 9,999 permutations). Visualization of the PCA was facilitated by ggplot2, incorporating 75% confidence ellipses to depict group clustering and variance.

To account for matching covariates, we also applied Procrustes analysis to test each healthy matched control and corresponding pre-CD's metabolomics variation across nested case control groups. Given the 1:4 nested case-control design, we randomly selected one matched control per pre-CD case to construct 1:1 matched subset and repeated this procedure four times to generate four independent datasets. Procrustes analysis was conducted using the *Procrustes* function of the vegan package[43]. Monte Carlo *p*-values for rotational agreement significance testing were determined from 9,999 permutations.

Survival analysis of metabolite-derived PCs with CD onset and CD related biomarkers

We investigated the association between metabolite derived principal components and the onset of Crohn’s disease (CD). This analysis involved fitting a conditional logistic regression model that takes into account the matching condition and adjusted for demographic factors including relation to proband (sibling or offspring) and CD-Multiplex family (>1 FDR with CD), using the “*clogit*” function from the survival package in R (version 3.5.5). The aim was to investigate the association between the top ten PCs explaining the most variance in the data derived from the metabolites and the future development of CD. A *p*-value less than 0.05 was considered significant.

Cross sectional analysis of metabolite-derived PCs with biomarkers of CD

We assess the correlation between metabolomic derived PCs and other CD-risk biomarkers, including CRP, FCP, gut barrier function makers, anti-microbial Antibodies, PCs derived from all bacterial taxa, and PCs derived from all measured proteins (See section above) using a partial Spearman correlation analysis account for nested group, relation to proband (sibling or offspring) and CD-Multiplex family (>1 FDR with CD) (*ppcor* package version 1.1). The Benjamini-Hochberg method was used to calculate *q*-values, with *q*-values lower than 0.05 considered significant.

Identification of independent metabolomic features

To reduce redundant information in the 1026 metabolites data we applied a Spearman correlation. The metabolomic analysis generated a total of 1,006 independent metabolites from 389 individuals (see above). A Spearman correlation values for each metabolites comparison that were less than 95% were included in the regression analysis aiming to identify metabolites associated with the future risk of CD. The regression employed the same conditional logistic regression model as mentioned above. The Benjamini-Hochberg procedure was applied to determine *q*-values. Q-values lower than 0.05 were considered statistically significant.

Assessment metabolites associated with future CD risk

We then identified CD risk-associated metabolites using conditional logistic regression. Out of 1006 independent metabolites, 68 exhibited significant association with future diagnosis of CD period (1.39 × 10^-4^< *q*-value <0.05) (Table S2). Out of these 68 CD-related metabolites, 14 were derived from imputation methods (with more than 10% imputation proportion, See methods). Thus, we further performed a sensitivity analysis consisting of restricting the analysis to samples with measured metabolites (i.e. removing the imputed value of metabolites with imputation proportions greater than 10%). Five out of these 14 metabolites were no longer significantly associated with CD onset, and we thus decided to exclude them from further analysis (Table S3).

Unsupervised clustering of pre-CD metabolites

Using partial Spearman correlations, we clustered pre-CD associated metabolites while accounting for the impact of demographic factors including relation to proband (sibling or offspring), CD-Multiplex family (>1 FDR with CD), and matching condition. A correlation coefficient threshold of *ρ* = | 0.6 | was set[44], and any pre-CD metabolites higher than this threshold were grouped into the same cluster.

**Supplementary Results**

***Metabolomic derived dimension reduction and Crohn’s disease risk***

The metabolomic analysis generated a total of 1026 serum metabolite profiles from 389 individuals. To reduce the dimension of the data, we first used PCA to analyze the high-dimensional metabolomic data in this cohort. We identified significant differences in the profiles of healthy matched control compared to pre-CD participants (fig. S7A, PERMANOVA; *p*-value = 1.0 × 10^-4^).

To better account for the nested case-control design, we then performed a Procrust analysis of pre-CD participants with each of the corresponding healthy matched controls. All four panels in fig. S7 B visualize the same global Procrustes analysis conducted on the full matched cohort (n = 78 pre-CD cases and 78 healthy controls). Each panel reflects a different random 1:1 pairing derived from the 1:4 matching design. These do not represent distinct biological subgroups, but rather repeated visualizations of the same dataset to demonstrate the robustness of the observed group-level differences. We found significant differences in the metabolomic profiles between pre-CD and healthy matched control (2.0 × 10^-4^ ≤ Monte Carlo *p*-value ≤ 0.02) . We then selected the top 10 metabolite derived principal components that contributed the most to the variance, which together explained 36.86% of the cumulative variance, to assess their association with the risk of CD onset. We found that PC4 (Odds ratio (OR) = 0.92, *p*-value = 0.004), PC5 (OR = 1.06, *p*-value = 0.029), and PC8 (OR = 1.07, *p*-value = 0.041) were significantly associated with future CD development (fig. S8).

***Metabolomic Principal components association with other Crohn’s disease risk biomarkers***

We performed a correlation analysis between metabolomic derived PCs and multiple biomarkers of CD risk as well as microbiome composition, and proteomic data derived PCs. We found the PC4, PC8 and PC5 principal components derived from metabolomic and associated with the risk of CD development (fig. S8) were positively correlated with baseline CRP (PC5: Spearman’s r=0.36, *q*-value = 3.52 × 10-13; PC8: = Spearman’s r=0.28, *q*-value = 1.98 × 10-8), a biomarker of systemic inflammation[45]. We also found a significant negative correlation between metabolomic-derived PC4 and LMR (n=359, Spearman’s r=-0.15, *q*-value =0.003). Interestingly, the metabolomic-derived PC4 was a protective factor for the onset of CD that was correlated with a decrease in LMR. We did not find significant correlations between CD-related metabolomic principal components with FCP, or serum antimicrobial antibody sum, or microbial diversity (Shannon index) (fig. S9). We found that other metabolomic derived principal components were significantly correlated with CRP, barrier function, anti-microbial antibodies sum, microbiome diversity, microbiome composition, and proteomic data. However, no correlation of metabolomic derived principal components was observed with antimicrobial antibodies or FCP.

***Correlation of metabolomic derived dimension reduction with microbiomics, proteomics derived dimension reduction***

We utilized a consistent dimensionality reduction method to extract the principal components (PCs) of the microbiome and proteome, selecting the top 10 PCs for each dataset to subsequently test for the association with serum metabolomic derived PCs. Among 379 subjects, six of the top 10 axes of metabolomic and microbiome PCs exhibited correlation (*p*-values < 0.05, but no *q*-values are less than 0.05.) (fig. S10), (metabolomic-derived PC4 vs. microbiota-derived PC2: Spearman’s r=0.16, *p*-value=0.002; metabolomic-derived PC5 vs. microbiota-derived PC4: Spearman’s r=0.15, *p*-value =0.004; metabolomic-derived PC8 vs. microbiota-derived PC1: Spearman’s r=-0.13, *p*-value =0.014). Compared to the other biomarkers used in this study, we observed the largest correlation coefficient when comparing serum metabolomic-derived PCs to proteomic-derived PCs. Notably metabolomic-derived PCs that were significantly associated with future onset of CD were correlated with proteomic-derived PCs (metabolomic-derived PC4 vs. proteomic-derived PC5: Spearman's r=0.43, *q*-value = 3.43 × 10-15; metabolomic-derived PC5 vs. proteomic-derived PC2: Spearman's r=0.43, *q*-value = 1.84 × 10-15; metabolomic-derived PC5 vs. proteomic-derived PC3: Spearman's r=0.39, *q*-value = 1.12 × 10^-12^, fig. S11).

We also identified metabolomic-derived PCs that were correlated with proteomic-derived PCs regardless of their association with CD onset (metabolomic-derived PC1 vs. proteomic-derived PC1: Spearman's r=-0.34, *q*-value=6.94 × 10^-10^; metabolomic-derived PC6 vs. proteomic-derived PC1: Spearman's r=-0.40, *q*-value=3.69 × 10^-13^; metabolomic-derived PC6 vs. proteomic-derived PC2: Spearman's r=-0.31, *q*-value =1.77 × 10^-8^, etc.)

***Unsupervised clustering revealing biological-related patterns of pre-CD significantly associated metabolites***

We assess if the 63 pre-CD associated metabolites were organized into separate cluster (Fig.1). Using a partial Spearman correlation we identified nine independent clusters of metabolites. The largest internal metabolite cluster consisted of eight metabolites, including two identified lipids: eicosenedioate (C20:1-DC), and 3beta,7alpha-dihydroxy-5-cholestenoate, as well as six unidentified associated metabolites. Another cluster was comprised of seven pre-CD metabolites, which included three xenobiotic related metabolites (p-cresol sulfate, 6-hydroxyindole sulfate, indolin-2-one), two acetylated peptides (phenylacetylcarnitine, phenylacetylglutamine), and two unknown metabolites. A separate cluster of seven pre-CD metabolites consisted solely of sphingomyelins-related metabolites. Four amino acids—3-methyl-2-oxovalerate, alpha-hydroxyisocaproate, 4-methyl-2-oxopentanoate, and 3-methyl-2-oxobutyrate—grouped together to form an internal metabolite cluster. Finally, there were five smaller, diverse internal metabolite cluster, each comprising only two different metabolites: N-acetylhistidine and N-acetyltyrosine from amino acid, glycylvaline and glu-gly-asn-val containing compound, Fibrinopeptide B and aspartate, ascorbate and isocitrate, quinolinate and an unidentified metabolite.

***Pre-CD associated metabolites are not confounded by other CD risk factors***

A sensitivity analysis assessed whether the association between the 63 pre-CD metabolites and future CD onset could be confounded by other biomarkers associated with risk of CD risk, including CRP, FCP, LMR, AS, and gut microbial diversity (Shannon index) (see Methods for details, Fig.2, Table S7-11). When we adjusted for LMR, 59 (93.7%) metabolites remained significantly associated with CD onset. When adjusted for AS, 62 (96.8%) metabolites except glu-gly-asn-val and 1-lignoceroyl-GPC (24:0), remained significant. When adjusted for CRP, 56 (90.5%) metabolites except phosphocholine, quinolinate, bilirubin (E,Z or Z,E), 1-lignoceroyl-GPC (24:0), N-acetylhistidine, and Fibrinopeptide B (1-9), remained significant. When adjusted for Shannon alpha diversity index all 63 (100%) metabolites remained significant. When we adjusted for FCP, 26 (41.3%) remained significantly associated with CD onset, including lysine metabolism, ascorbate (Vitamin C), indolin-2-one, monoacylglycerol, Sphingomyelins, hexosylceramides (HCER) and lactosylceramides (LCER). Summarizing the results above, 26 (41.2%) pre-CD metabolites maintained their significance after adjustments were made for each potential confounder individually.

**Supplementary Information**

**Supplementary Notes**

**Supplementary Note 1 (PDF):** Subject Screening & Demographics questionnaire.

**Supplementary Note 2 (PDF):** Control Subject Screening & Enrollment questionnaire.

**Supplementary Note 3 (PDF):** Confirmation of New Crohn’s Disease questionnaire.


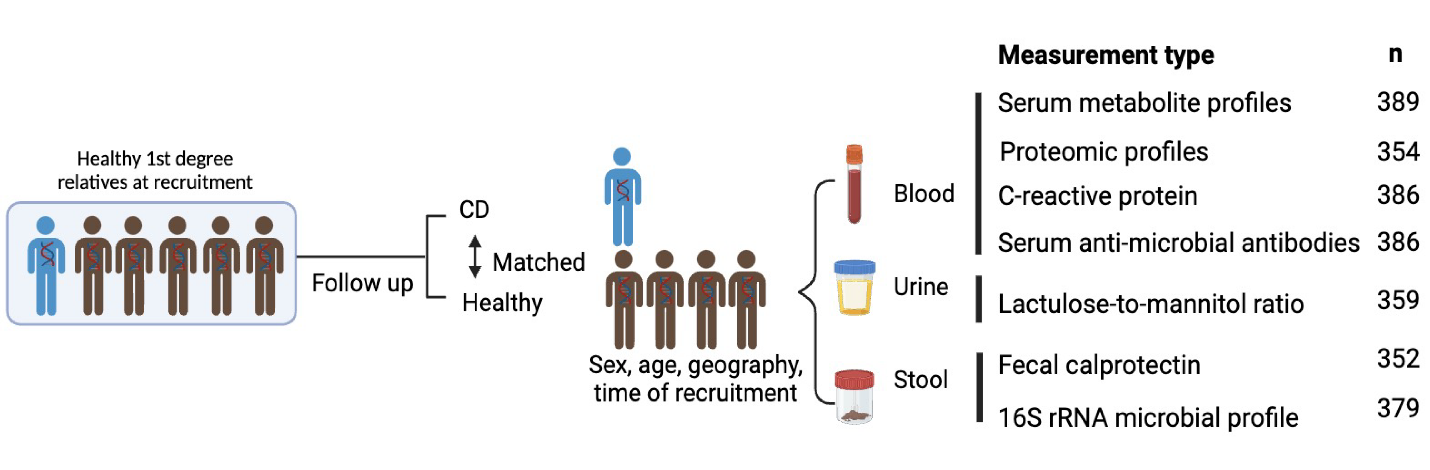


**Fig. S1. Study design and samples size of each dataset included in this analysis**


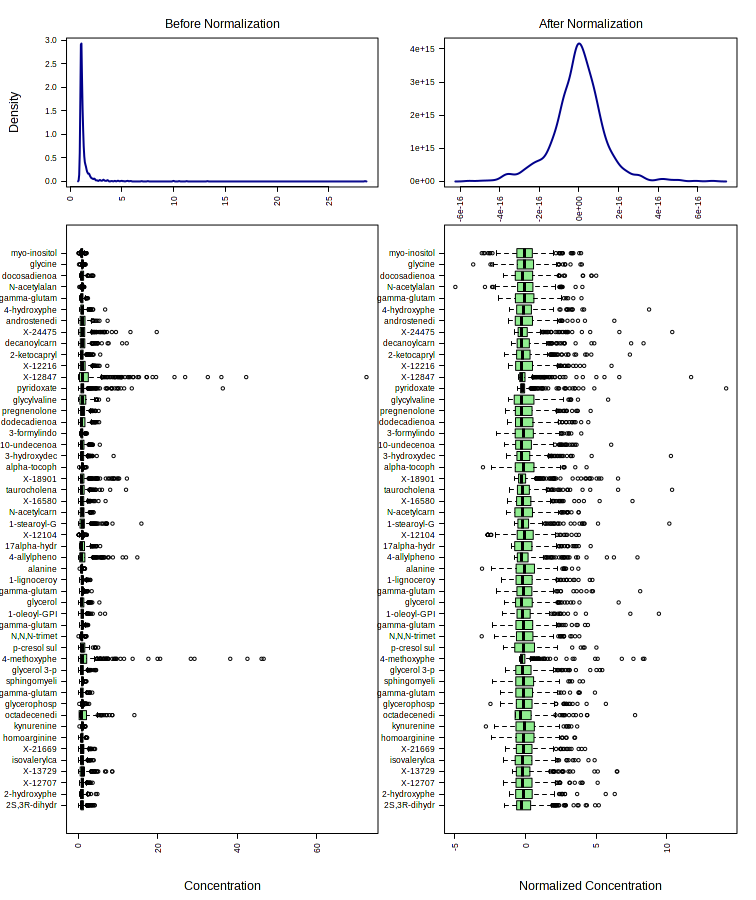


**Fig. S2. Distributions of serum metabolites before and after normalization.** The Box plots and kernel density plots were generated before and after autoscaling. We restricted the boxplots to 50 random metabolomic features. The kernel density plots are based on all samples (n=389). Feature names include known metabolites and unknowns labeled with “X-” identifiers.

**
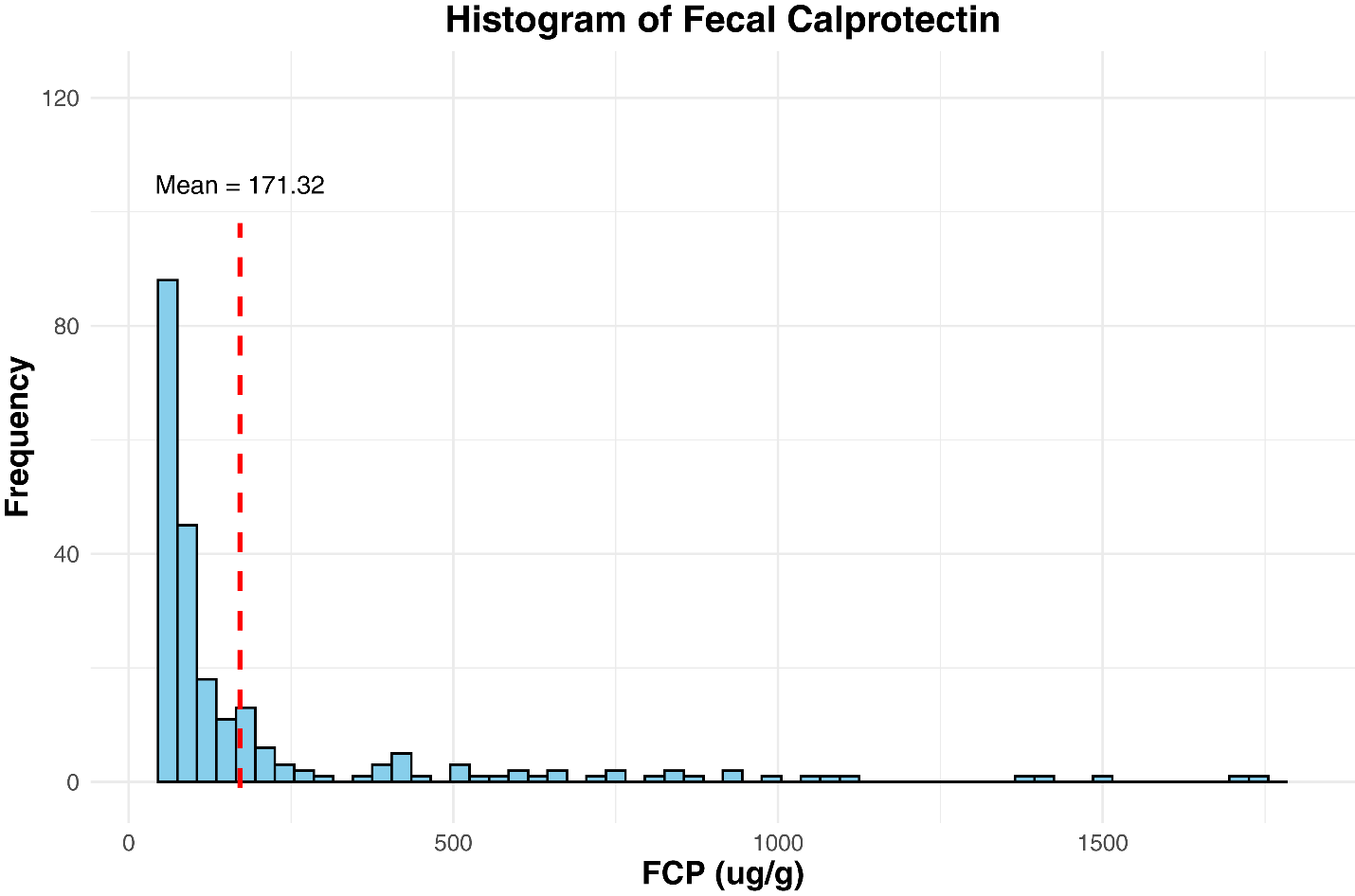
** **Fig. S3. Histogram of fecal calprotectin distribution (n= 352).** The red vertical line represents the mean value of Winsorized fecal calprotectin. The x-axis represents the bins of fecal calprotectin values, and the y-axis indicates the number of participants within each bin.


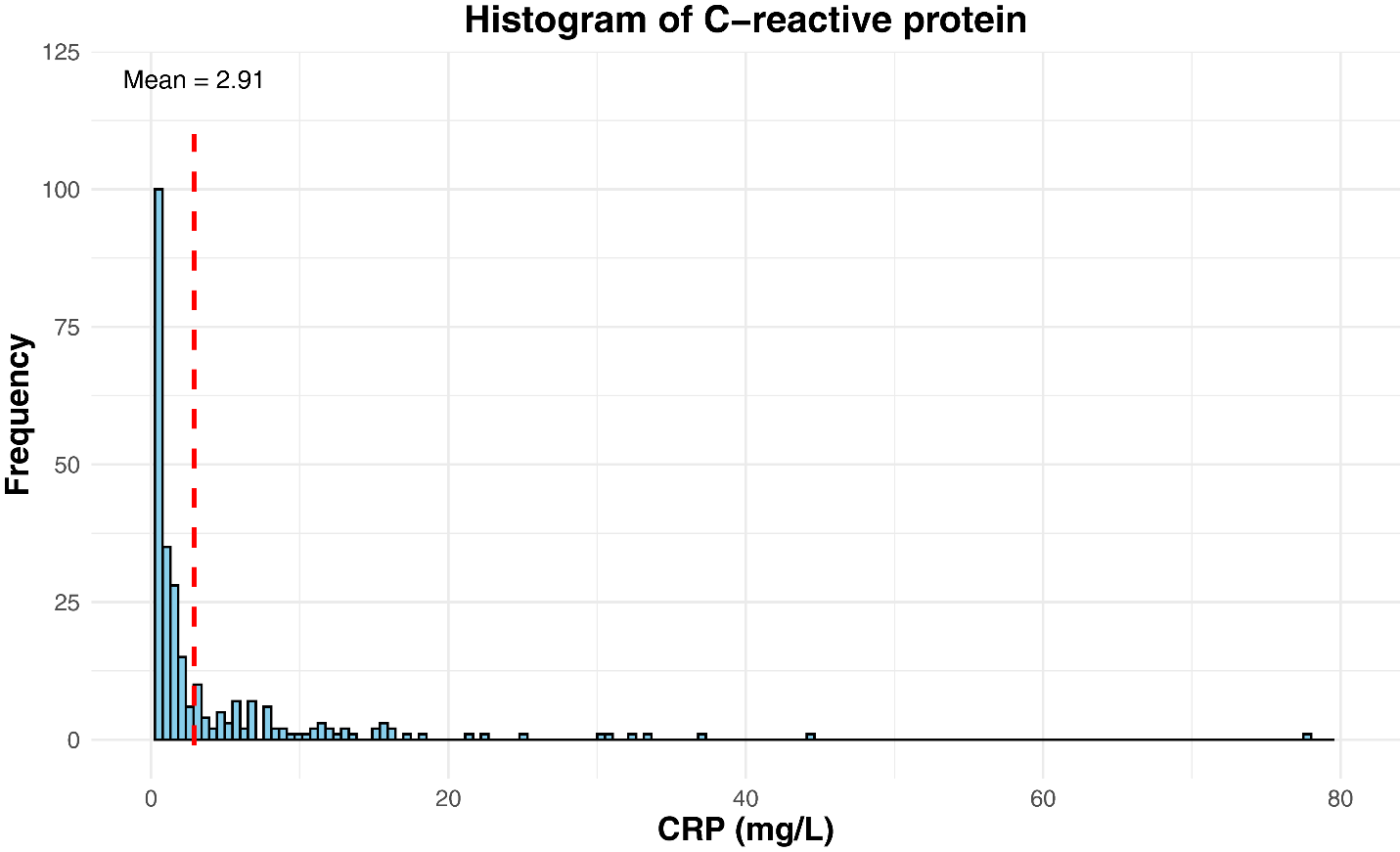
 **Fig. S4.** **Histogram of C-reactive protein distribution (n= 386).** The red vertical line represents the mean value of C-reactive protein. The x-axis represents the bins of C-reactive protein values, and the y-axis indicates the number of participants within each bin. CRP, C- reactive protein.

**
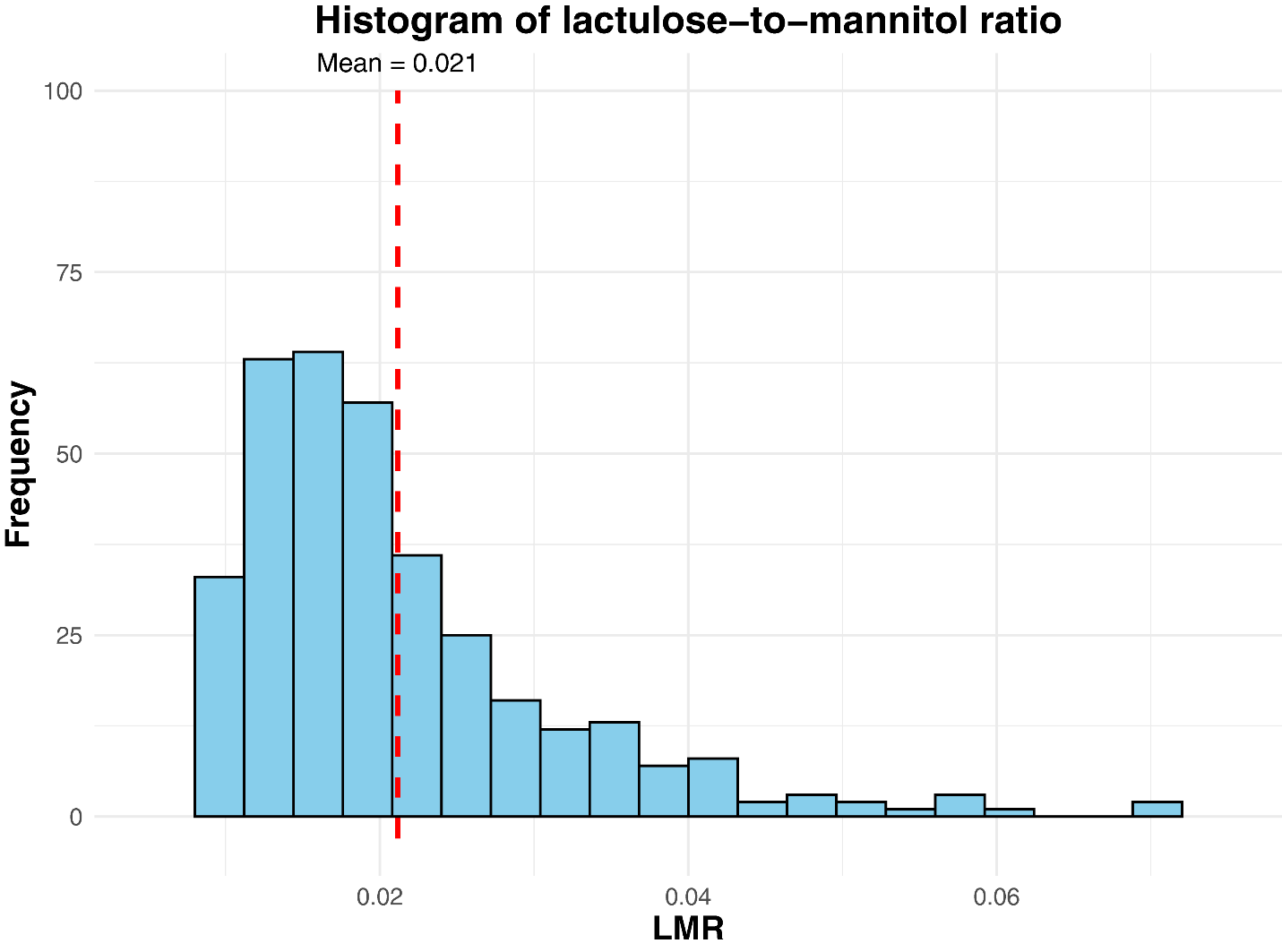
**

**Fig. S5. Histogram of Lactulose-to-mannitol ratio distribution (n= 359).** The red vertical line represents the mean value of Lactulose-to-mannitol ratio distribution. The x-axis represents the bins of LMR values, and the y-axis indicates the number of participants within each bin. LMR, Lactulose-to-mannitol ratio.


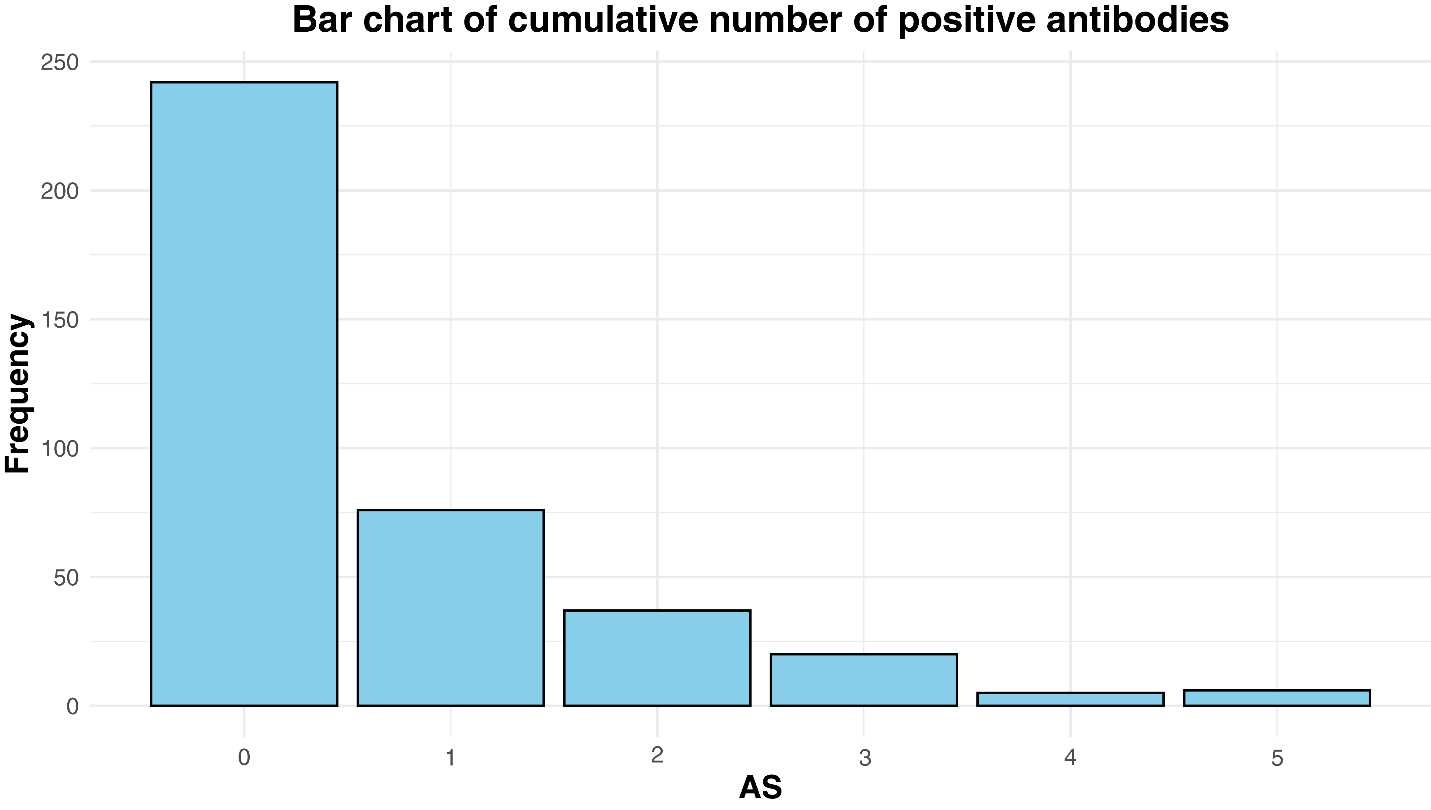


**Fig. S6. Histogram antimicrobial antibodies sum distribution (n= 386).** The x-axis represents the number of positive antimicrobial antibodies values (See Material and methods), and the y-axis indicates the number of participants within each bin. AS, the cumulative number of positive antimicrobial antibodies.

**
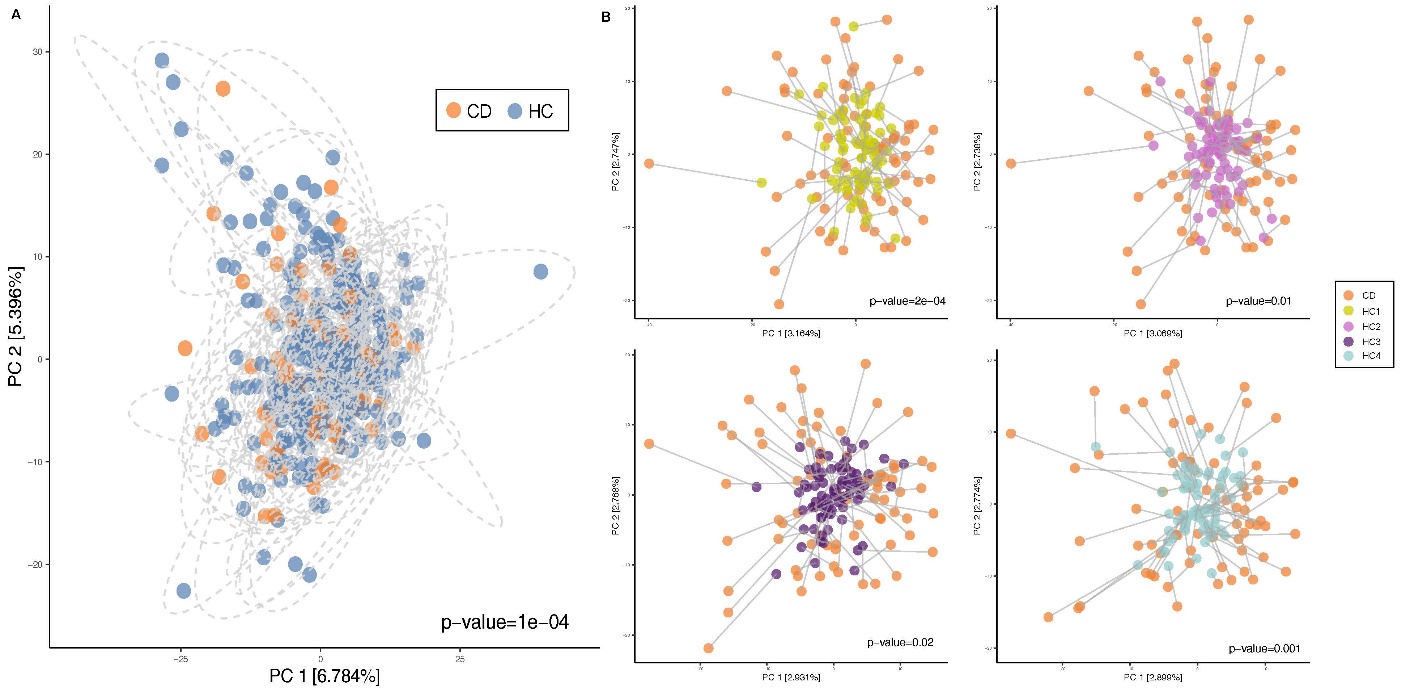
**

**Fig.S7. Serum metabolic profile is associated with risk of Crohn’s disease.**

**(A)** Principal Component Analysis of all participants (n=78 preclinical CD; n=311 HMC) based on serum metabolite profiles. Permutational Multivariate Analysis of Variance (PERMANOVA) on the full model revealed a significant overall group difference (*p*-value=0.0001, based on Euclidean distances of all dimensions). Gray ellipses represent 75% confidence intervals around each group. **(B**) Procrustes analysis was used to evaluate the overall dissimilarity in metabolomic profiles between pre-CD individuals (n = 78) and HMCs under a 1:4 matching design. In each of the four subpanels, one HMC was randomly selected from the four matched controls for each pre-CD case to create a 1:1 matched subset for comparison. Orange dots represent pre-CD individuals, and colored dots represent the selected HMCs in that comparison. Each gray line connects a pre-CD case to its matched HMC. The *p*-values indicate the statistical significance of the overall dissimilarity between the two groups in each matched subset. Metabolites were autoscaling-transformed for comparability purposes. CD: Crohn’s disease; HMC: healthy match control.

**
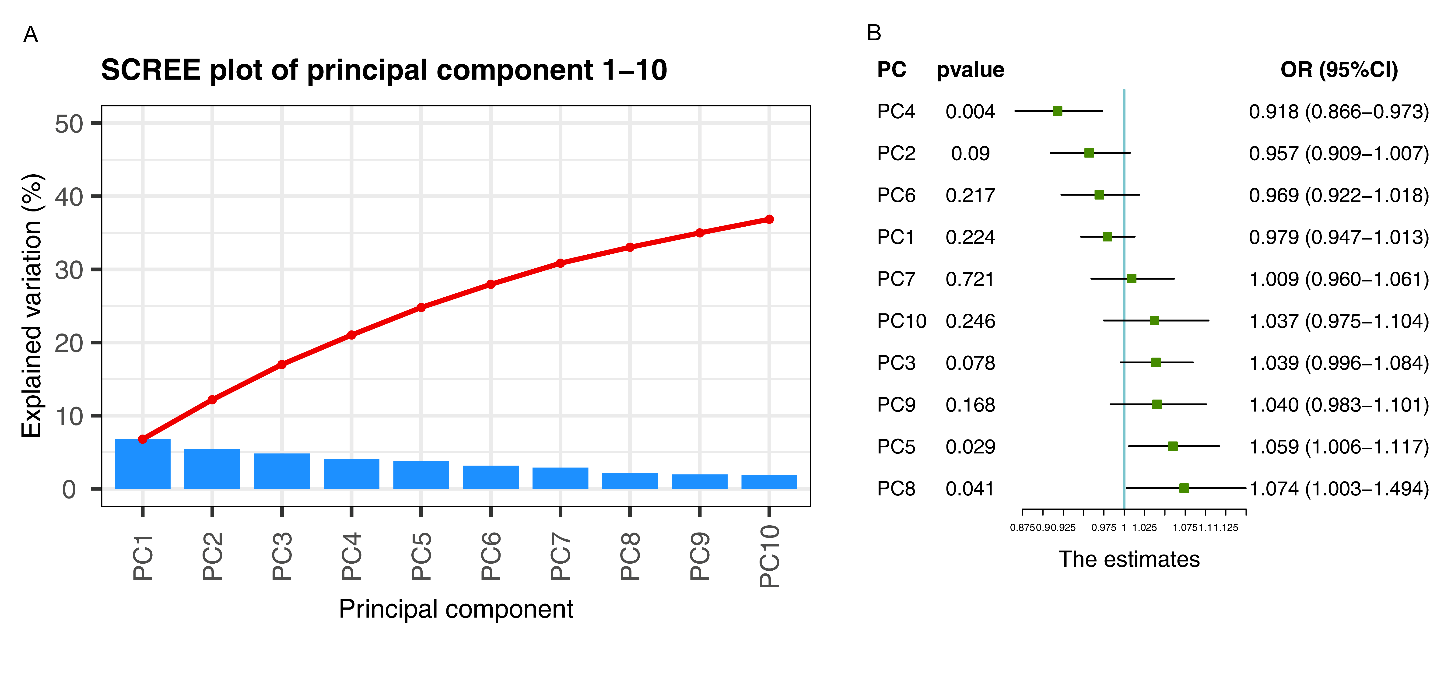
**

**Fig. S8. Serum metabolomic principal components are associated with future Crohn’s disease onset.** Unsupervised dimensionality reduction was applied to the 1026 identified serum metabolites using principal component analysis (see Methods). (A) Scree plot of the top 10 principal components (x-axis) and the y-axis the explained variance of each principal components. The red lines show the cumulative overall explained variance. (B) The coefficient plot of the estimates (x-axis) of each of the top 10 serum metabolic principal components (y-axis) for their association with future CD onset using conditional logistic regression and adjusted for CD-multiplex family, and relation to proband (sibling vs offspring). Estimates and 95% CI (x-axis) for the top 10 metabolic PCs (y-axis). CD, Crohn’s disease; PC, principal component.

**
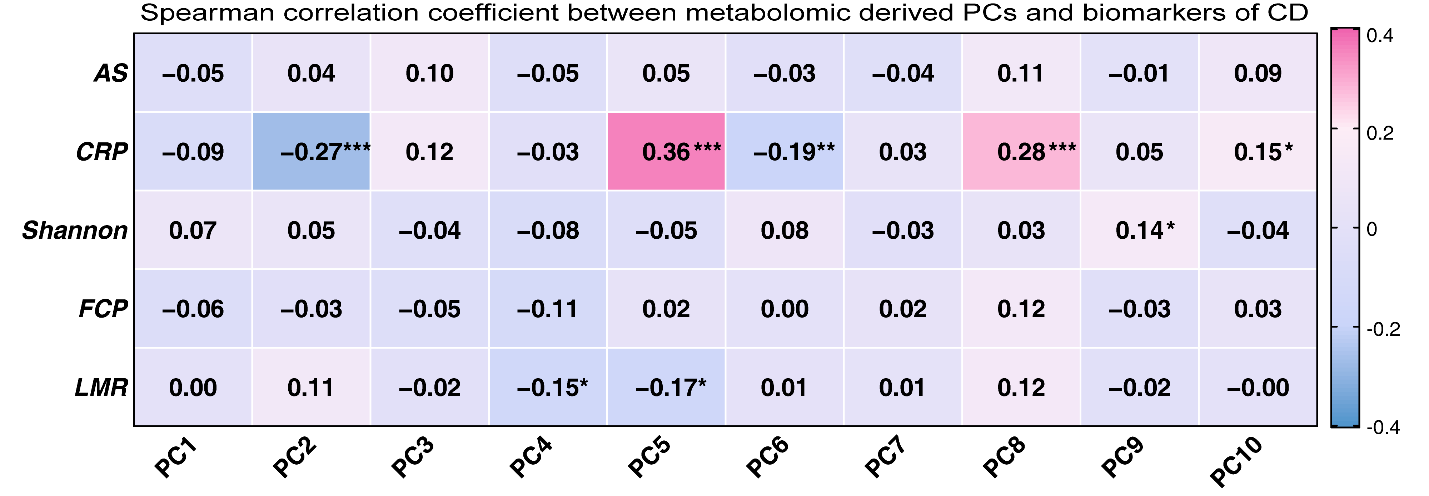
**

**Fig. S9.** **Heatmap illustrating the correlation coefficient between the top 10 principal components derived from serum metabolomic and antimicrobial antibodies sum, C-reactive protein, fecal calprotectin, and microbiome Shannon index of alpha diversity.** Partial Spearman correlation analysis was adjusted for the matching group (age, sex assigned at birth, time of recruitment, and geography), relation proband (sibling or offspring), and CD-Multiplex family. CD: Crohn’s disease, AS: antimicrobial antibodies sum, FCP: fecal calprotectin, CRP: C-reactive protein, LMR: lactulose to mannitol ratio. Stars indicated significant q-values calculated using the Benjamini-Hochberg (**q-*value ≤ 0.05, ***q-*value ≤ 0.01, ****q-*value ≤ 0.001). Pink indicates a positive correlation coefficient while blue indicates a negative correlation coefficient.

**
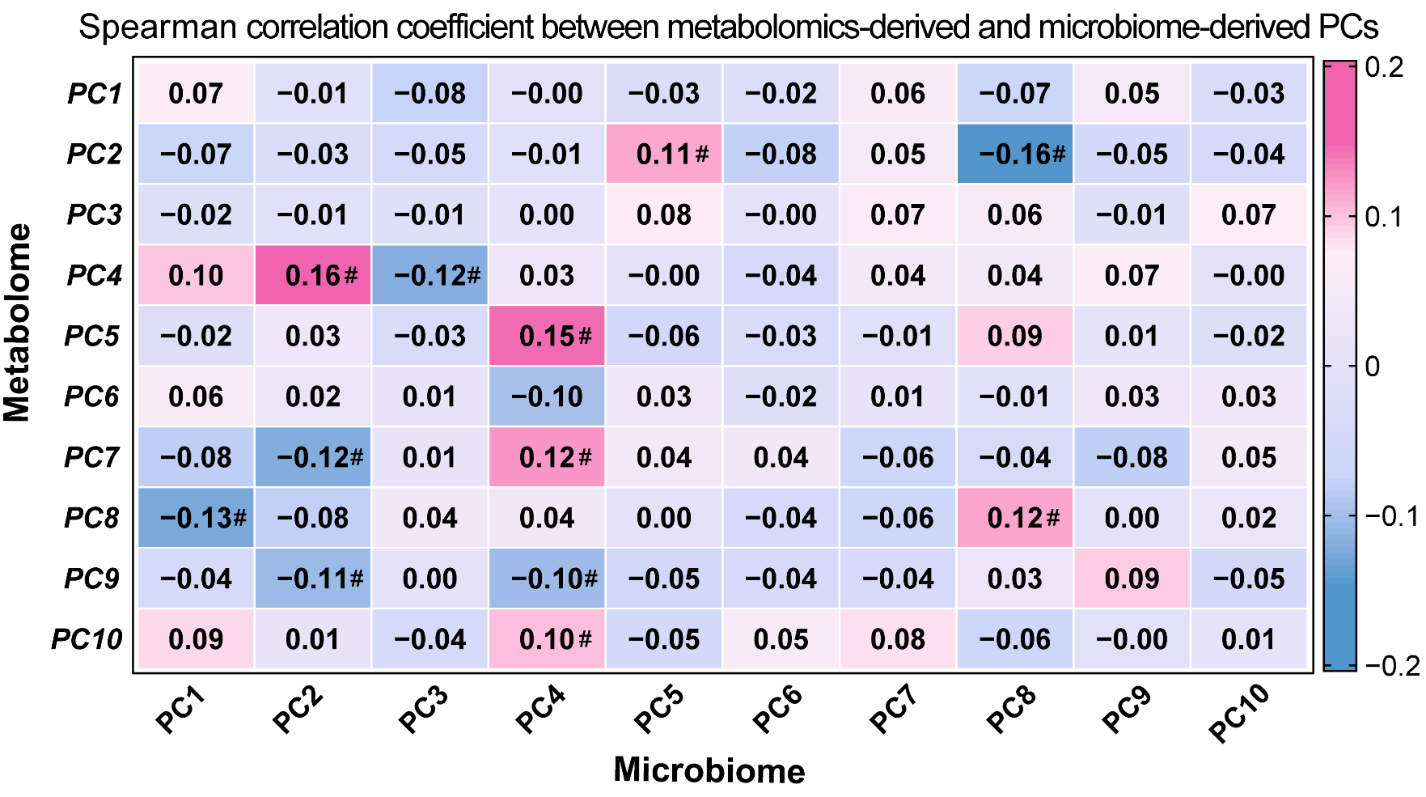
**

**Fig. S10.** **Heatmap illustrating the correlation between the top 10 principal components derived from serum metabolomic and the top 10 principal components derived from 16s rRNA sequencing analyzed at the genus level (415 genera) of the taxonomy.** Partial Spearman correlation analysis was adjusted for the matching group (age, sex assigned at birth, time of recruitment, and geography), relation proband (sibling or offspring), and CD-Multiplex family. CD: Crohn’s disease. ^#^ *p-*value < 0.05. Pink indicates a positive correlation coefficient while blue indicates a negative correlation coefficient.


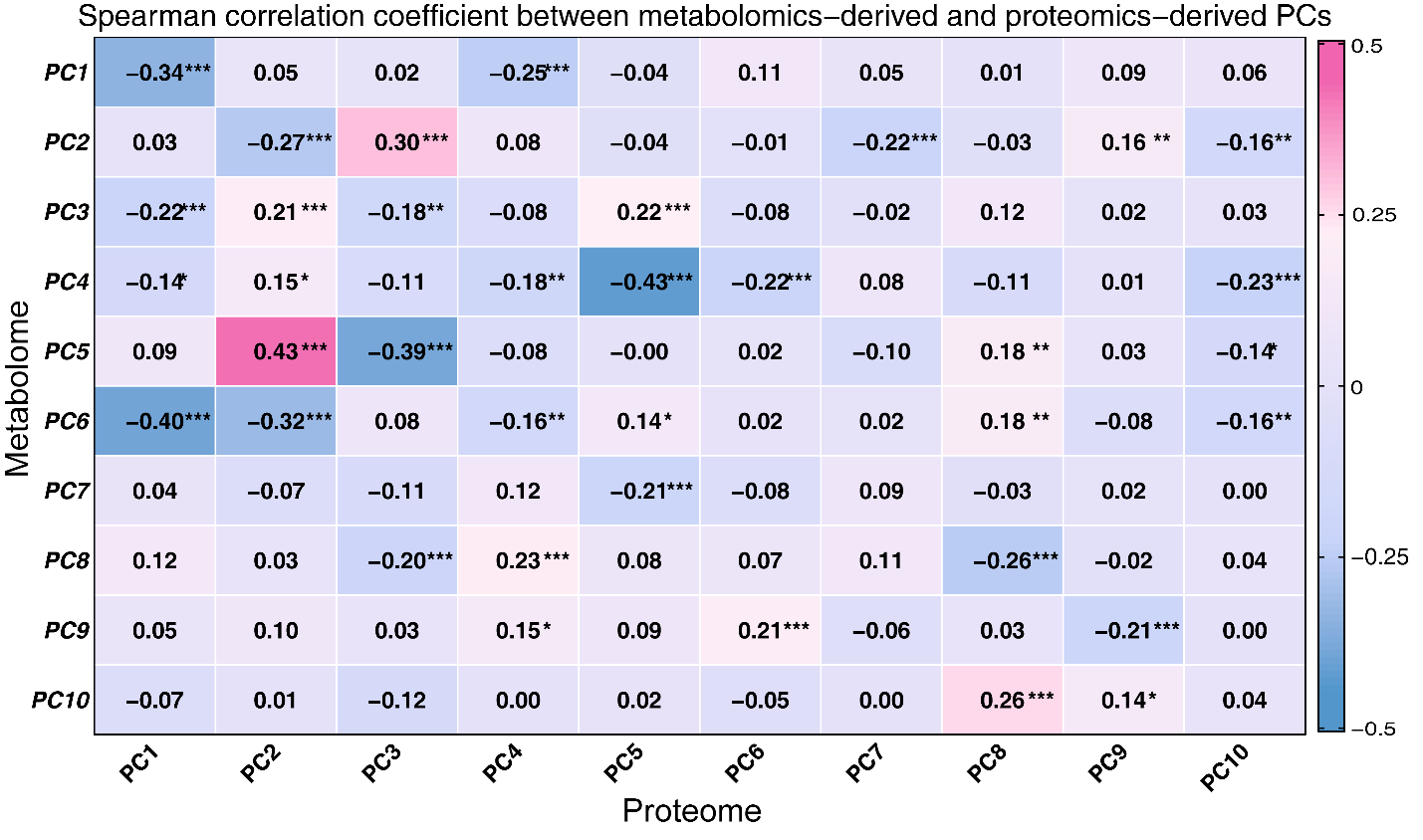


**Fig. S11.** **Heatmap illustrating the correlation between the top 10 principal components derived from serum metabolomic and the top 10 principal components derived from the serum proteomic (446 proteins).** Partial Spearman analysis was adjusted for the matching group (age, sex assigned at birth, time of recruitment, and geography), relation proband (sibling or offspring), and CD-Multiplex family. CD: Crohn’s disease. Stars indicated q-values calculated using the Benjamini-Hochberg (**q*-value ≤ 0.05, ** *q*-value ≤ 0.01, *** *q*-value ≤ 0.001). Pink indicates a positive correlation coefficient while blue indicates a negative correlation coefficient.


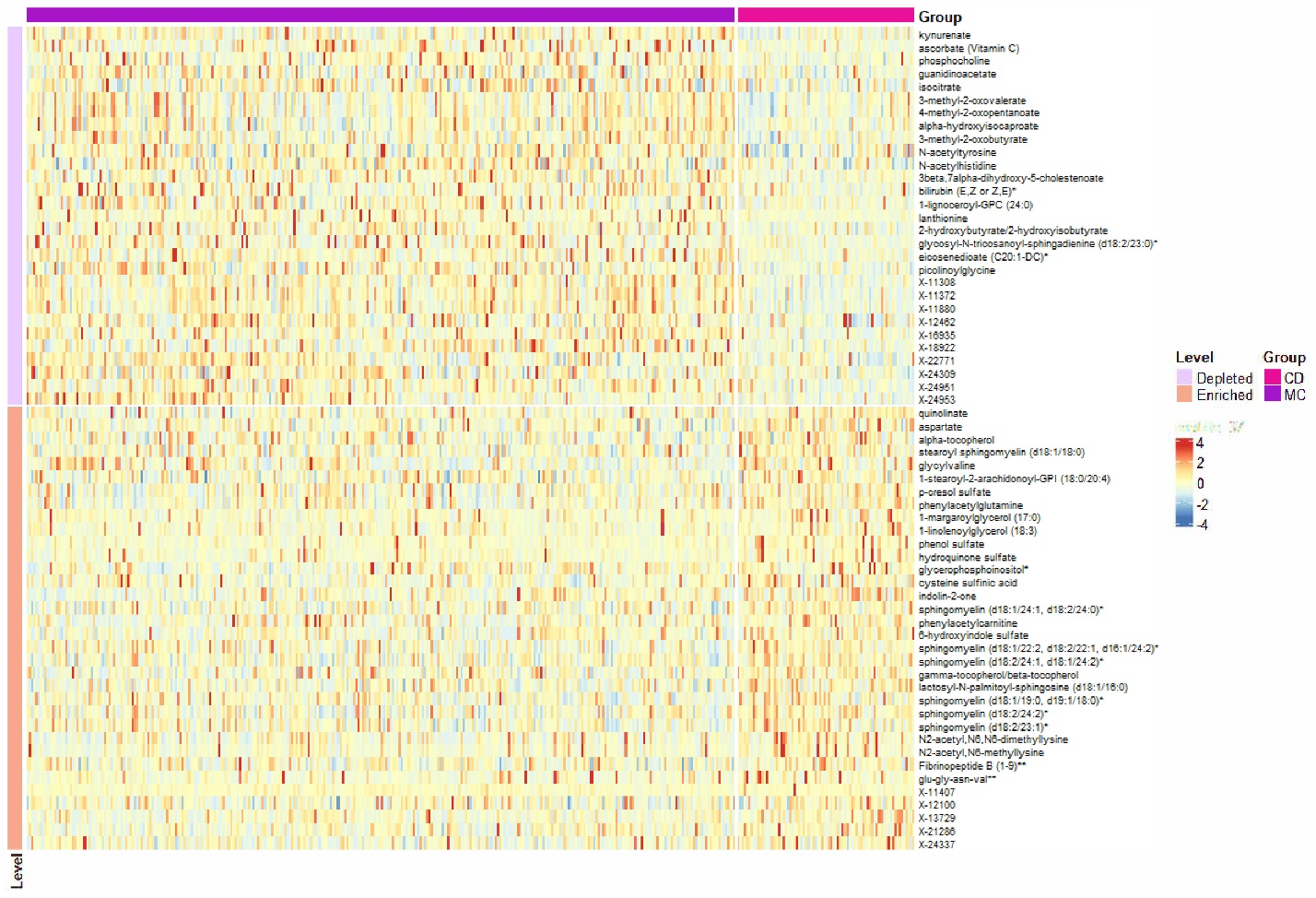


**Fig. S12. Heatmap of the 63 serum metabolites associated with CD onset in the study cohort.** This heatmap visualizes the log-transformed concentrations of metabolites (Y-axis) across different samples (X-axis). The data were normalized by scaling each row to have a mean of zero and a standard deviation of one, facilitating the comparison of metabolomic patterns across the cohort. The color intensity in each cell reflects the relative amount of the metabolite in that sample, with the scale ranging from -4 (blue) to 4 (red). Rows annotations (on the left) indicate the level of each metabolite (depleted or enriched in pre-CD). Column annotations (on the top) represent the outcome groups of the samples (pre-CD or healthy matched control). ** Indicates a compound putatively identified by Metabolon Inc. through matching to its proprietary spectral libraries and orthogonal analytical evidence without a chemical standard；* Indicates a compound putatively characterized to a chemical class based on spectral similarity using Metabolon Inc.’s annotation protocols, pending confirmation by a standard. CD: Crohn’s disease. X- indicates unknown but defined compounds.

**
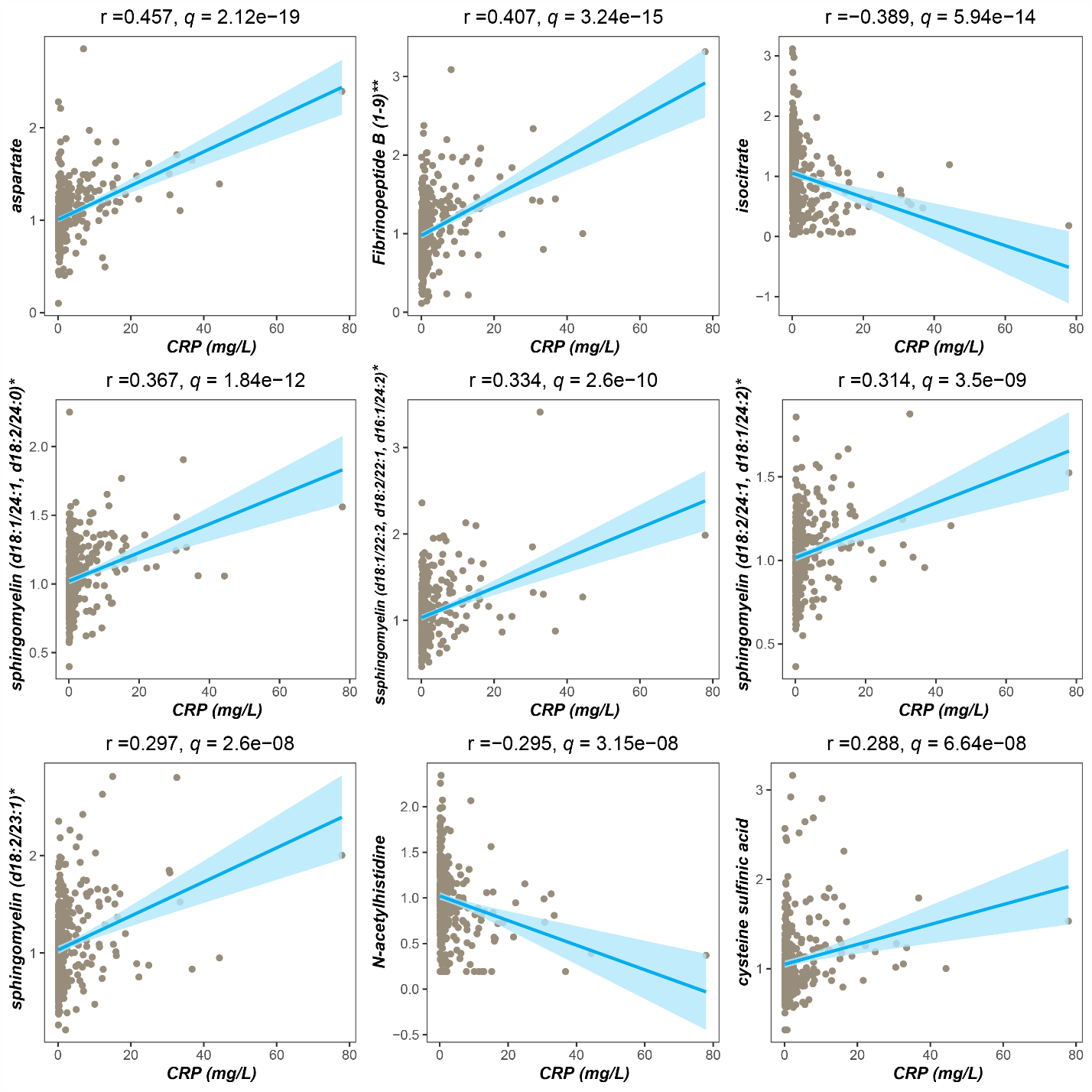
**

**Fig. S13****. Scatter plot of pre-CD metabolites correlation with c-reactive protein.** Scatter plot of the nine most significant correlations (based on *q*-value) between pre-CD serum metabolites and C-reactive protein. The Y-axis represents the arbitrary unit of the given metabolites (after group normalization, See Methods). The X-axis represents the C-reactive protein value (mg/L) measured at recruitment. The correlation coefficient was calculated using partial Spearman analysis accounting for the matching group (age, sex assigned at birth, time of recruitment, and geography), relation proband (sibling or offspring), and CD-Multiplex family. The *q*-*values* were calculated using the Benjamini-Hochberg. Each dot represents the value of a given individual. The blue line represents the best regression line based on the two variables of the samples, while the blue region indicates the 95% confidence interval of the best regression line. * Indicates a compound putatively characterized to a chemical class based on spectral similarity using Metabolon Inc.’s annotation protocols, pending confirmation by a standard. CRP, c-reactive protein; CD, Crohn’s disease.

**
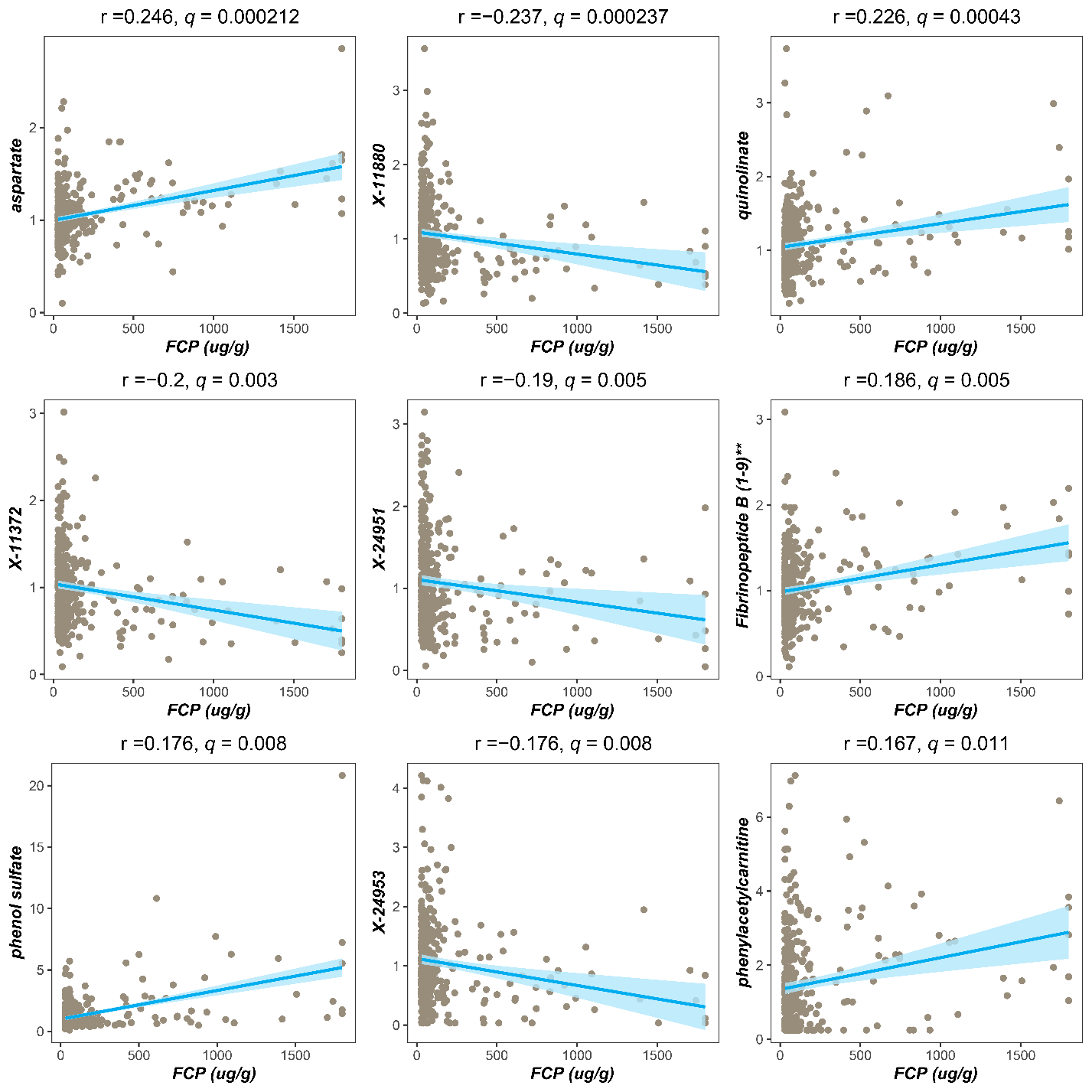
**

**Fig. S14.** **Scatter plot of pre-CD metabolites correlation with fecal calprotectin.** Scatter plot of the nine most significant correlations (based on *q*-value) between pre-CD serum metabolites and FCP. The Y-axis represents the arbitrary unit of the given metabolites (after group normalization, See Methods). The X-axis represents the FCP in 30-1800 mg/L measured at recruitment. The correlation coefficient was calculated using the partial Spearman analysis accounting for the matching group (age, sex assigned at birth, time of recruitment, and geography), relation proband (sibling or offspring), and CD-Multiplex family. The *q*-values were calculated using the Benjamini-Hochberg. Each dot represents the value of a given individual. The blue line represents the best regression line based on the two variables of the samples, while the blue region indicates the 95% confidence interval of the best regression line. ** Indicates a compound putatively identified by Metabolon Inc. through matching to its proprietary spectral libraries and orthogonal analytical evidence without a chemical standard; FCP: fecal calprotectin; CD, Crohn’s disease. X- indicates unknown but defined compounds.

**
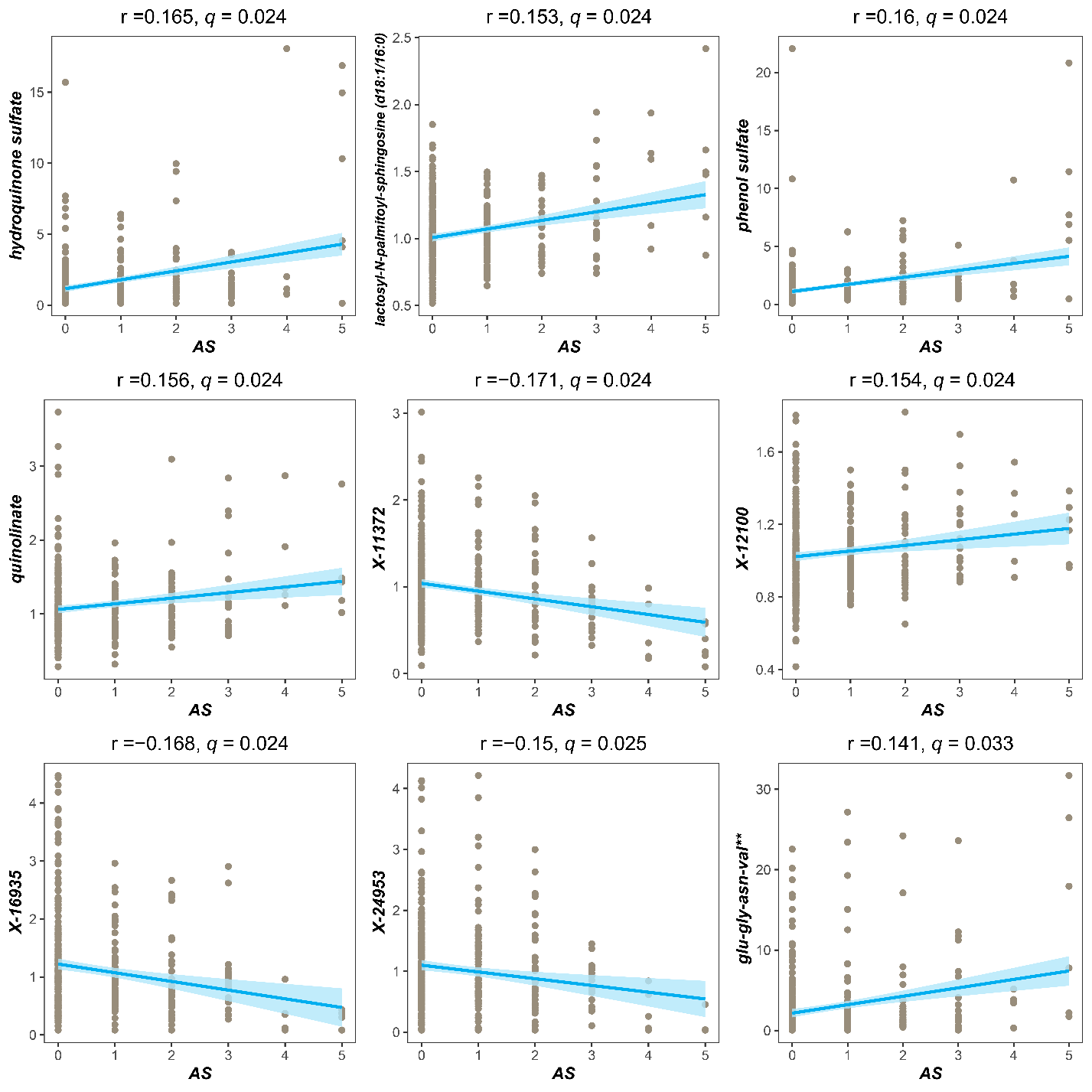
**

**Fig. S15.** **Scatter plot of pre-CD metabolites correlation with antimicrobial antibody sum.** Scatter plot of the nine most significant correlations (based on *q*-value) between pre-CD serum metabolites and antimicrobial antibody sum. The y-axis represents the arbitrary unit of the given metabolites (after group normalization, See Methods). The x-axis represents the antimicrobial antibody sum from 0 to 5 indicating the total number of positive antibody responses (See methods). The correlation coefficient was calculated using the partial Spearman analysis accounting for the matching group (age, sex assigned at birth, time of recruitment, and geography), relation proband (sibling or offspring), and CD-Multiplex family. The *q-values* were calculated using the Benjamini-Hochberg. Each dot represents the value of a given individual. The blue line represents the best regression line based on the two variables of the samples, while the blue region indicates the 95% confidence interval of the best regression line. ** Indicates a compound putatively identified by Metabolon Inc. through matching to its proprietary spectral libraries and orthogonal analytical evidence without a chemical standard. AS: antibody sum; CD: Crohn’s disease. X- indicates unknown but defined compounds


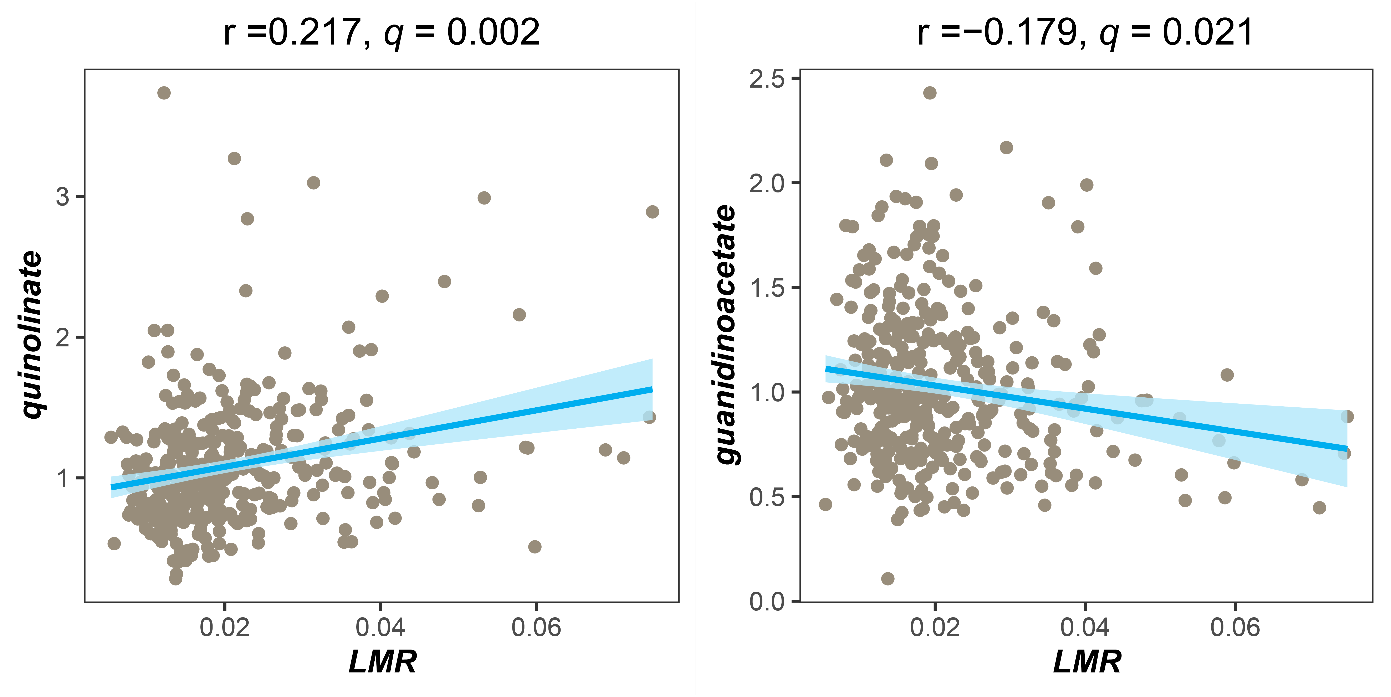
**Fig. S16.** **Scatter plot of pre-CD metabolites correlation with lactulose-to-mannitol ratio.** The significant correlations (*q*-value<0.05) between pre-CD serum metabolites after group normalization (see methods) (y-axis) and LMR in 0-0.1 units (x-axis) measured at recruitment. Partial Spearman analysis adjusted for the matching group (age, sex assigned at birth, time of recruitment, and geography), relation proband (sibling or offspring), and CD-Multiplex family. The *q*-values were calculated using the Benjamini-Hochberg. Each dot represents the value of a given individual. The blue line represents the best regression line based on the two variables of the samples, while the blue region indicates the 95% confidence interval of the best regression line. LMR: lactulose-to-mannitol ratio; CD, Crohn’s disease.


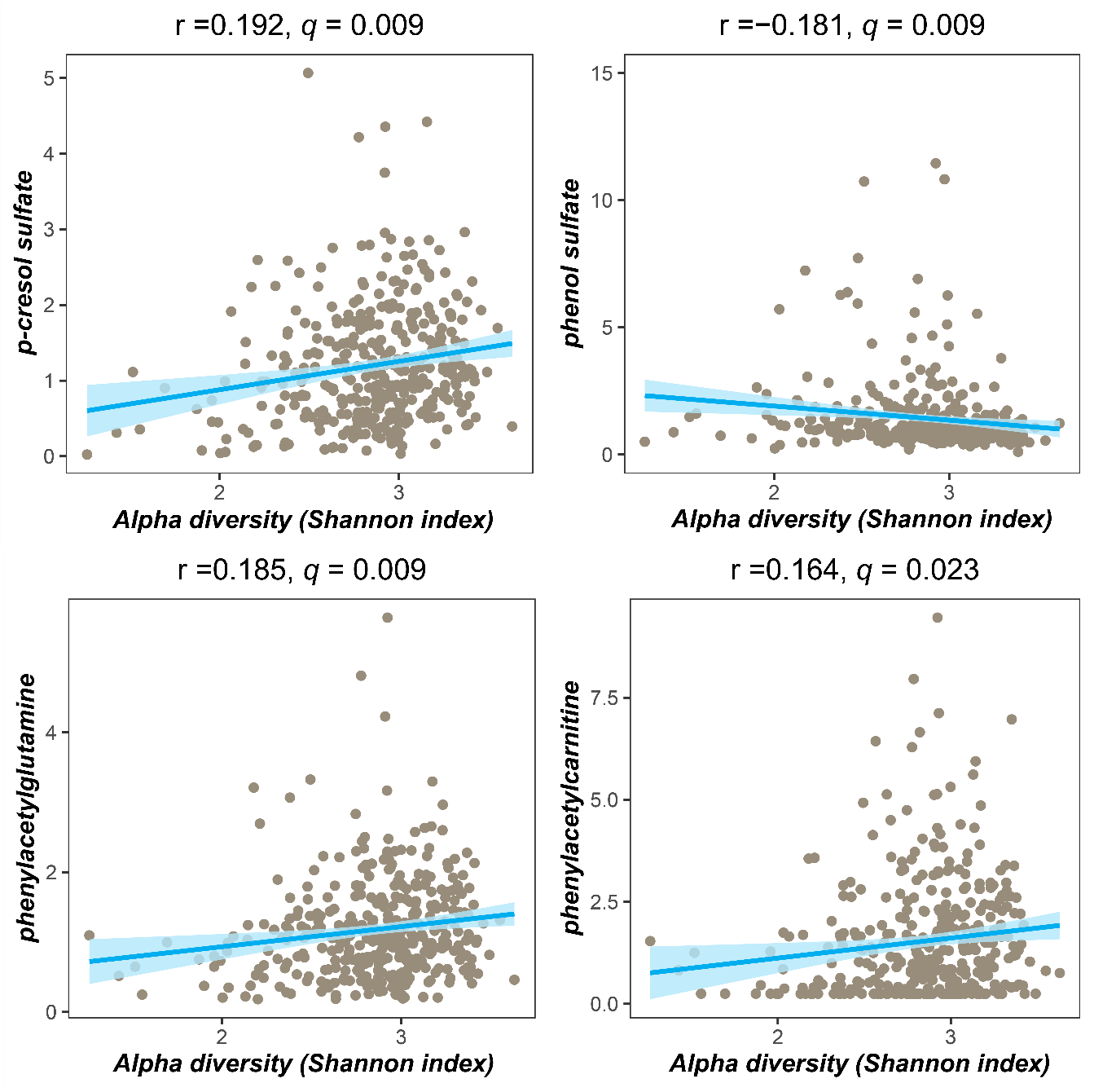


**Fig. S17.** **Scatter plot of pre-CD metabolites correlation with Shannon alpha diversity index.** The significant correlations (*q*-value<0.05) between pre-CD serum metabolites after group normalization (see methods) (y-axis) and alpha diversity measure by the Shannon index in 1-4 units (x-axis) measured at recruitment. Partial Spearman analysis adjusted for the matching group (age, sex assigned at birth, time of recruitment, and geography), relation proband (sibling or offspring), and CD-Multiplex family. The *q*-values were calculated using the Benjamini-Hochberg. Each dot represents the value of a given individual. The blue line represents the best regression line based on the two variables of the samples, while the blue region indicates the 95% confidence interval of the best regression line. CD, Crohn’s disease.


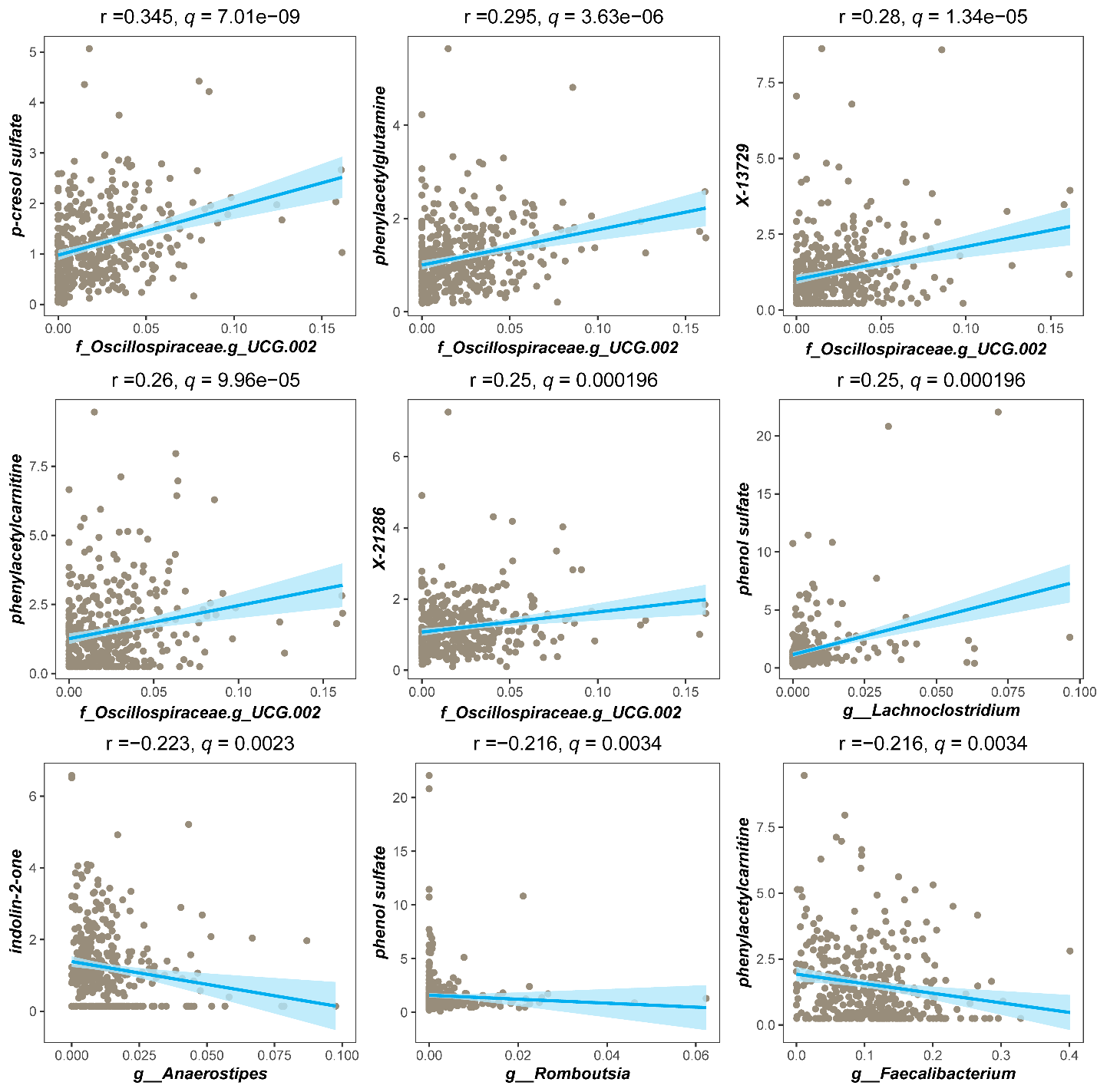


**Fig. S18.** **Scatter plot of pre-CD metabolites correlation with microbial genera relative abundance.** The nine most significant correlations (based on q-value) between pre-CD serum metabolites after group normalization (see methods) (x-axis) and relative abundance of gut microbial taxa (y-axis), measured at recruitment. Partial Spearman analysis adjusted for the matching group (age, sex assigned at birth, time of recruitment, and geography), relation proband (sibling or offspring), and CD-Multiplex family were performed. The *q*-values were calculated using the Benjamini-Hochberg. Each dot represents the value of a given individual. The blue line represents the best regression line based on the two variables of the samples, while the blue region indicates the 95% confidence interval of the best regression line. X- indicates unknown but defined compounds. 'g_' represents the genus level of taxonomy. 'f_' represents the family level of taxonomy; CD, Crohn’s disease.


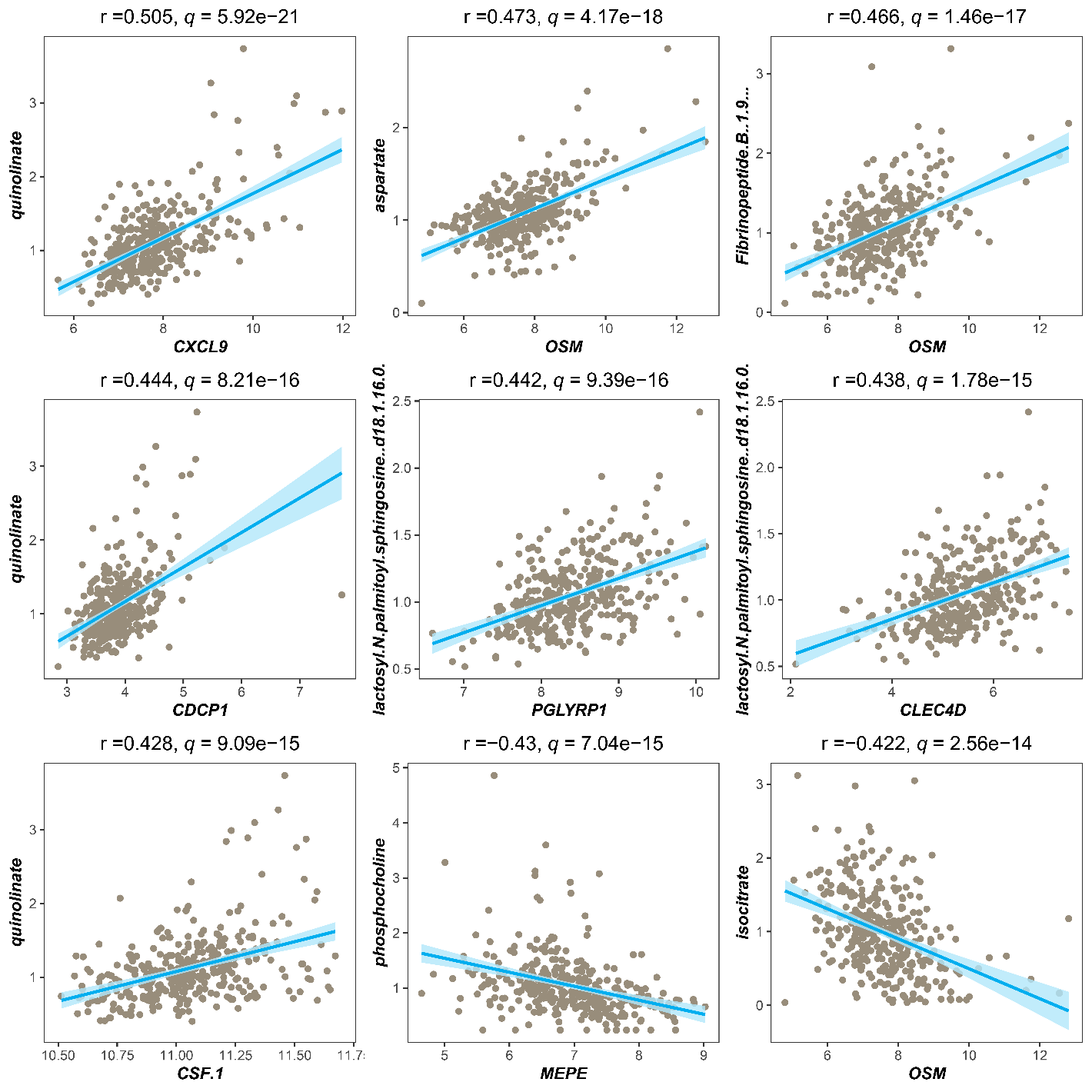


**Fig. S19.** **Scatter plot of pre-CD metabolites correlation with serum proteins.** The nine most significant correlations (based on *q*-value) between pre-CD serum metabolites after group normalization (see methods) (x-axis) and normalized protein level (y-axis) measured at recruitment are displayed. Partial Spearman analysis adjusted for the matching group (age, sex assigned at birth, time of recruitment, and geography), relation proband (sibling or offspring), and CD-Multiplex family. The *q*-values were calculated using the Benjamini-Hochberg. Each dot represents the value of a given individual. The blue line represents the best regression line based on the two variables of the samples, while the blue region indicates the 95% confidence interval of the best regression line. CD, Crohn’s disease.


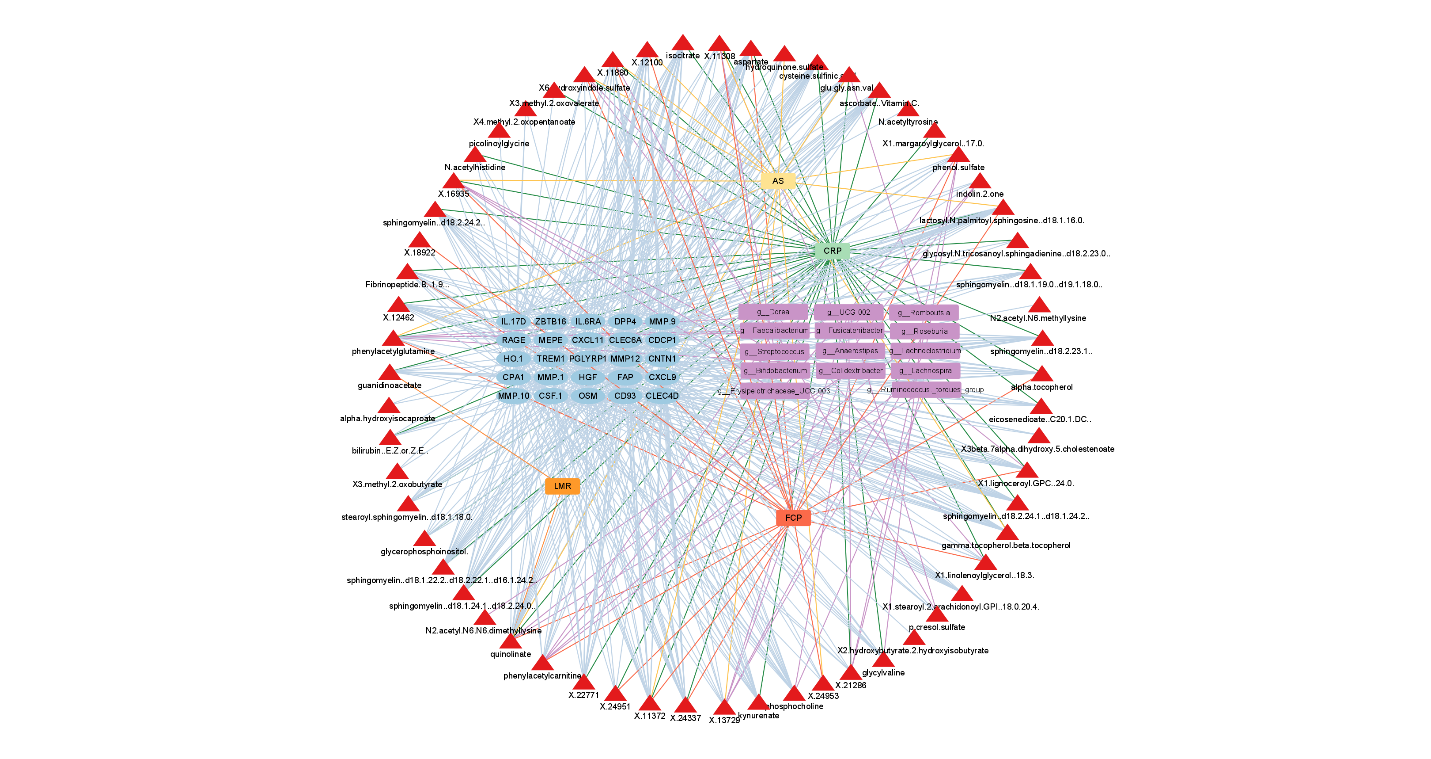


**Fig. S20. Integrative network analysis of pre-CD serum metabolites and other pre-CD biomarkers.** This network highlights significant correlations (*q*-value < 0.05) derived consistently with the findings from Figure 3. Pre-CD-associated serum metabolites (red triangles) and previously reported CD risk biomarkers (rectangles). Blue rectangles represent 23 serum proteins, orange indicates LMR, red denotes FCP, green represents CRP, yellow corresponds to AS, and purple signifies 20 genera. Line thickness reflects the significance of the correlations (based on *q*-value). CD: Crohn’s disease; FCP: fecal calprotectin; LMR: lactulose-to-mannitol ratio; CRP: C-reactive protein; AS: serum antimicrobial antibody sum. Feature names include known metabolites and unknowns labeled with “X-” identifiers.

**Supplementary Tables**

**Supplementary Table 1:** Number of samples used in each analysis

**Supplementary Table 2:** Univariable conditional logistic regression results for the association between serum metabolites and the future onset of Crohn's disease

**Supplementary Table 3:** Logistic regression results for the association between the non-imputed portions of pre-CD metabolites and the future onset of Crohn's disease

**Supplementary Table 4:** List of the 63 serum metabolites remaining significantly associated with the future onset of Crohn’s disease after excluding metabolites with imputed data in more than 10% of the cohort

**Supplementary Table 5:** Univariable conditional logistic regression results of shorter follow up duration group for serum metabolites associated with future onset of Crohn’s disease

**Supplementary Table 6:** Univariable conditional logistic regression results of longer follow up duration group for serum metabolites associated with future onset of Crohn’s disease

**Supplementary Table 7:** Results of the targeted univariate conditional logistic regression model for the 63 serum metabolites associated with future onset of Crohn’s disease after adjusting for Lactulose to Mannitol ratio

**Supplementary Table 8:** Results of the targeted univariate conditional logistic regression model for the 63 serum metabolites associated with future onset of Crohn’s disease after adjusting for c-reactive protein

**Supplementary Table 9:** Results of the targeted univariate conditional logistic regression model for the 63 serum metabolites associated with future onset of Crohn’s disease after adjusting for serum anti-microbial antibody sum

**Supplementary Table 10:** Results of the targeted univariate conditional logistic regression model for the 63 serum metabolites associated with future onset of Crohn’s disease after adjusting for gut microbial diversity (Shannon index)

**Supplementary Table 11:** Results of the targeted univariate conditional logistic regression model for the 63 serum metabolites associated with future onset of Crohn’s disease after adjusting for fecal calprotectin

**Supplementary Table 12:** Partial spearman correlation coefficient of 63 serum metabolites associated with future onset of Crohn’s disease and c-reactive protein after accounting for the matching condition (age, sex, country, and follow up duration), CD-multiplex family, and relation to proband (sibling vs offspring)

**Supplementary Table 13:** Partial spearman correlation coefficient of 63 serum metabolites associated with future onset of Crohn’s disease and fecal calprotectin after accounting for the matching condition (age, sex, country, and follow up duration), CD-multiplex family, and relation to proband (sibling vs offspring)

**Supplementary Table 14:** Partial spearman correlation coefficient of 63 serum metabolites associated with future onset of Crohn’s disease and serum anti-microbial antibody sum after accounting for the matching condition (age, sex, country, and follow up duration), CD-multiplex family, and relation to proband (sibling vs offspring)

**Supplementary Table 15:** Partial spearman correlation coefficient of 63 serum metabolites associated with future onset of Crohn’s disease and Lactulose to Mannitol ratio after accounting for the matching condition (age, sex, country, and follow up duration), CD-multiplex family, and relation to proband (sibling vs offspring)

**Supplementary Table 16:** Partial spearman correlation coefficient of 63 serum metabolites associated with future onset of Crohn’s disease and gut microbial diversity (Shannon index) after accounting for the matching condition (age, sex, country, and follow up duration), CD-multiplex family, and relation to proband (sibling vs offspring)

**Supplementary Table 17:** Partial spearman correlation coefficient of 63 serum metabolites associated with future onset of Crohn’s disease and 20 microbial genera after accounting for the matching condition (age, sex, country, and follow up duration), CD-multiplex family, and relation to proband (sibling vs offspring)

**Supplementary Table 18:** Partial spearman correlation coefficient of 63 serum metabolites associated with future onset of Crohn’s disease and 23 proteins after accounting for the matching condition (age, sex, country, and follow up duration), CD-multiplex family, and relation to proband (sibling vs offspring)
