# Supplementary material for: Metabolomics reveal distinct molecular pathways associated with future risk of Crohn’s Disease
Source: Gut Microbes. 2025 Sep 5;17(1):2546998. doi: 10.1080/19490976.2025.2546998 (PMC12416195; doi:10.1080/19490976.2025.2546998)
Supplement: Supplementary_Note_2.docx [file KGMI_A_2546998_SM8952.docx]

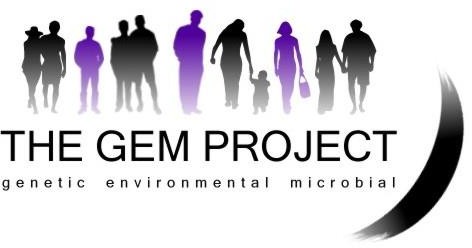


Document # 7

Control Subject Screening & Enrollment

| **Subject ID:** | – – | |
| --- | --- | --- |
| **Control For Subject:** | – – | |
| **Assignment Date:** | / /  Year Month Day |  |
| **Follow-up Date:** | / / | **Time:** |
|  | Year Month Day |  |

| Sample Type | Barcode Label |  |
| --- | --- | --- |
| **Urine** | (Affix label here) | Volume (ml) |
| **Stool** | (Affix label here) |  |
| **Blood** | (Affix label here) |  |

To be completed by the Research Assistant or Research Coordinator.

This questionnaire contains confidential and private personal health information and must be kept in the secure locked office of the Regional Coordinator.

Part 1 – Health Status Review

| 1. Are you in your usual good health today? | Yes | No |  |
| --- | --- | --- | --- |
| 2. Are you currently taking antibiotics or have you taken antibiotics in the past 30 days? | Yes | No | *IF YES follow-up with subject 30 days after the last dose of antibiotics* |
| 3. Have you taken antibiotics in the past 3 months? | Yes | No | *If NO go to Q.5* |
| 4. If yes, which antibiotic(s)? | 1. | |  |
|  | 2. | |  |
| 5. Have you taken antibiotics in the past 6 months? | Yes | No | *If NO go to Q.7* |
| 6. If yes, which antibiotic(s)? | 1. | |  |
|  | 2. | |  |
| 7. Have you taken antibiotics in the past 12 months? | Yes | No | *If NO go to Q.9* |
| 8. If yes, which antibiotic(s)? | 1. | |  |
|  | 2. | |  |
| 9. Have you ever been diagnosed with any chronic or recurring gastro-intestinal disease or bowel disease? | Yes | No | *IF YES go to Q.10*  *IF NO go to Q.11 or Q.15* |
| 10. What type of bowel disease do you have? |  | |  |
| ***Questions 11 – 14 are for adult subjects only (16 years of age and over). For minors proceed to Q.15*** | | | |
| 11. Have you recently and unintentionally lost weight? | Yes | No | *If NO go to Q.16* |
| 12. If yes, how much have you lost? | (kg) | |  |
| 13. Was this weight loss planned? | Yes | No |  |
| 14. Was the unintentional weight loss in the last 3 months more than 15% of your baseline weight? | Yes | No | *If YES subject is NOT ELIGIBLE. Please complete Doc # 5 – Subject Demographic & Health Review. Go to Q.16* |
| ***Question 15 is for pediatric subjects only (less than 16 years old)*** | | | |
| 15. Were you or your parents ever told by your doctor that your rate of growth (height) was abnormal? | Yes | No | *Check growth chart and re-consider eligibility.* |
| 16. Are you pregnant? | Yes | No | *If YES subject is NOT ELIGIBLE If NO go to Q.18* |
| 17. If yes, what is the approximate due date? | / /  Year Month Day | | *Follow-up with subject 6 months after giving birth.* |
| 18. Do you have belly pain more than once a week? (Unrelated to menstruation)? | Yes | No | *If YES subject is NOT ELIGIBLE.*  *Please complete Doc #5 – Subject Demographic & Health Review* |

| 19. Has this belly pain occurred more than once per week for longer than 3 months in the past year? | Yes | No |  | *If YES subject is NOT ELIGIBLE. Please complete Doc #5 – Subject Demographic & Health Review* |
| --- | --- | --- | --- | --- |
| 20. Do you have liquid diarrhea more than 3 times per day? | Yes | No |  | *If YES subject is NOT ELIGIBLE. Please complete Doc #5 – Subject Demographic & Health Review.*  *If NO go to Q.22* |
| 21. Has the diarrhea (> 3 times per day) been occurring for more than 3 months in the last year? | Yes | No |  | *If YES subject is NOT ELIGIBLE. Please complete Doc #5 – Subject Demographic & Health Review* |
| 22. Do you have blood in your stool with most stools? | Yes | No |  | *If YES subject is NOT ELIGIBLE. Please complete Doc #5 – Subject*  *Demographic & Health Review* |
| 23. Are you experiencing any illness or infection today? | Yes | No |  |  |
| 24. **Subject is eligible as a control subject?** | Yes | No |  | *If NO go to Q.25* |
| 25. If no, has Doc #5 – “Subject Demographic & Health Review” been completed? | Yes | No | N/A |  |

Part 2 – Administrative

| Completed On: | / /  Year Month Day |  |
| --- | --- | --- |
| Completed by: |  |  |
|  | Print Name | Signature |
| Designation: | Research Coordinator Principal Investigator | |
| PI Confirmation: |  |  |
|  | Print Name | Signature |

The information on this form should be registered in the online database within 72 hours of completion. Please record the date of completion and the SUBJECT ID of the control on each page.

| Submission Date: | / /  Year Month Day |
| --- | --- |
