# Supplementary material for: Metabolomics reveal distinct molecular pathways associated with future risk of Crohn’s Disease
Source: Gut Microbes. 2025 Sep 5;17(1):2546998. doi: 10.1080/19490976.2025.2546998 (PMC12416195; doi:10.1080/19490976.2025.2546998)
Supplement: Supplementary_Note_1.docx [file KGMI_A_2546998_SM8951.docx]

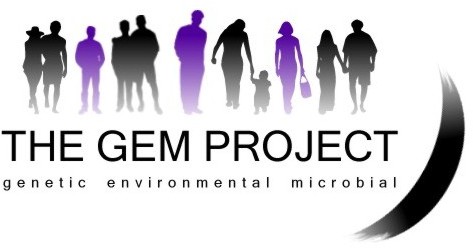


Document # 2

Subject Screening & Demographics Questionnaire

**Subject ID:**  – –

**Enrollment Date:** / /

Year Month Day

**Follow-up Date:** / / **Time:**

Year Month Day

| Sample Type | Barcode Label |  |
| --- | --- | --- |
| **Urine** | (Affix label here) | Volume (ml) |
| **Stool** | (Affix label here) |  |
| **Blood** | (Affix label here) |  |

**To be completed by the Research Assistant or Research Coordinator.**

**This questionnaire contains confidential and private personal health information and must be kept in the secure locked office of the Regional Coordinator.**

**Instructions – Please Read Carefully**

Please ensure that consent forms are signed before proceeding with the assessment and sample collection.

Consent Signed: Signed by:
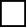
 Subject
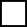
 Mother
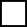
 Father
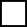
 Guardian Assent Signed:
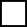
 Yes
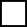
 No
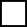
 Not Applicable

Consent Version Number:

This questionnaire is to be completed by research staff.

The questions are designed to provide the research team with the necessary details for subject enrollment into the study. The questions will confirm eligibility.

It is imperative that the subject be declared “**disease free**” to be eligible for this study.

If you have any questions, please review the **Manual of Operations** and/or the **Standards of Practice**, or contact the National Project Office.

**Part 1 – Subject Information**

Last Name: First Name:

Middle Name:

Sex:
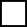
 Male
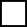
 Female Relation to Proband:
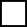
 Brother
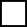
 Sister
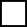
 Offspring

*If the subject is less than 6 years old, or greater than 35 years old, then the*

Date of Birth: / / Age:

Year Month Day

*subject IS NOT ELIGIBLE for the study.*

| Enrollment Date: | / / | Subject is a Minor: | Yes | No |
| --- | --- | --- | --- | --- |
|  | Year Month Day | (Under 16 yrs of age) |  |  |

# Contact Information:

If the subject is a minor, please fill out the names and telephone numbers of the parents or guardian.

# Name Telephone

Mother: ( ) - Father: ( ) - Guardian: ( ) -

**Telephone/Address for**:
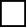
 Subject, or (if the subject is a minor)
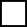
 Mother
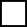
 Father
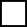
 Guardian

| Residence: | ( ) - | Address |  |
| --- | --- | --- | --- |
| Business: | ( ) - |  |  |
|  | Ext: |  |  |
|  |  | City/Town: |  |
| Other: | ( ) - | Province: |  |
|  | Ext: | Postal Code: |  |
|  |  | Email Address: |  |

# Caregiver Information:

Please provide the name and office number of the subject’s family physician or pediatrician, and the GI specialist treating the Proband.

# Name Office Telephone

Family Physician: ( ) -

Other Physician: ( ) -

**Part 2 – Health Review**

1. Are you in your usual good health today?
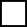
 Yes
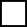
 No
2. Are you currently taking antibiotics or have you taken antibiotics in the past 30 days?

Yes
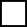
 No *IF YES follow-up with subject 30*

*days after the last dose of*

*antibiotics*

1. Have you taken antibiotics in the past 3 months?
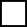
 Yes
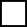
 No *IF NO go to Q.5*
2. If yes, which antibiotic(s)? 1.

2.

1. Have you taken antibiotics in the past 6 months?
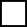
 Yes
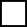
 No *IF NO go to Q.7*
2. If yes, which antibiotic(s)? 1.

2.

1. Have you taken antibiotics in the past 12 months?
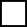
 Yes
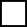
 No *IF NO go to Q.9*
2. If yes, which antibiotic(s)? 1.

2.

1. Have you ever been diagnosed with any chronic or recurring gastro-intestinal disease or bowel disease?

Yes
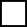
 No *IF YES go to Q.10*

*IF NO go to Q.11 or Q.15*

1. What type of bowel disease do you have?

*Questions 11 – 14 are for adult subjects only (over 16 years of age). For minors proceed to Q.15*

1. Have you recently and unintentionally lost weight?
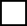
 Yes
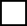
 No *If NO go to Q.16*
2. If yes, how much have you lost? (kg)
3. Was this weight loss planned?
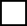
 Yes
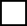
 No
4. Was the unintentional weight loss in the last 3 months more than 15% of your baseline weight?

Yes
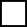
 No *If YES subject is NOT ELIGIBLE*

*If NO go to Q.16*

*Question 15 is for pediatric subjects only (less the 16 years old)*

1. Were you or your parents ever told by your doctor that your rate of growth (height) was abnormal?

Yes
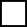
 No *Check growth chart and re-*

*consider eligibility.*

1. Are you pregnant?
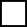
 Yes
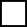
 No *If NO go to Q.18*
2. If yes, what is the approximate due date? / /

Year Month Day

*Follow-up with subject 6 months after giving birth.*

1. Do you have belly pain more than once a week? (unrelated to menstruation)?

Yes
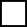
 No *If NO go to Q.20*

1. Has this belly pain occurred more than once per week for longer than 3 months in the past year?

Yes
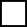
 No *If YES subject is NOT ELIGIBLE*

1. Do you have liquid diarrhea more than 3 times per day?

Yes
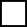
 No *If NO go to Q.22*

1. Has the diarrhea (> 3 times per day) been occurring for more than 3 months in the last year?

Yes
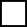
 No *If YES subject is NOT ELIGIBLE*

1. Do you have blood in your stool with most stools?
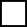
 Yes
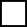
 No *If YES subject is NOT ELIGIBLE*
2. Are you experiencing any illness or infection today?
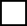
 Yes
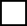
 No

**Part 3 – Eligibility Assessment**

Subject is declared full sibling or offspring of proband
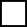
 Yes
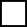
 No *If No subject is NOT ELIGIBLE*

Subject is between 6 and 35 years old
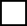
 Yes
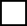
 No *If No subject is NOT ELIGIBLE*

Subject is currently pregnant
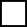
 Yes
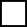
 No *If YES follow-up with subject 6 months after birth.*

Subject is diagnosed with diabetes
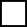
 Yes
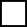
 No *If YES subject is NOT ELIGIBLE*

Subject is diagnosed with any of:

Celiac disease

Inflammatory bowel disease (IBD) Irritable bowel syndrome (IBS)

Yes
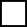
 No *If YES subject is NOT ELIGIBLE*

Subject presents significant symptoms of GI disease

(Please refer to health review above)

Subject is currently taking antibiotics or has taken antibiotics in the past 30 days

Yes
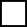
 No *If YES subject is NOT ELIGIBLE*

Yes
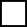
 No *If YES follow-up with subject 30*

*days after the last dose of*

*antibiotics*

**Part 4 – Administrative**

Written Registration Completed:

/ /

Year Month Day

Completed by:

Print Name Signature

Designation:
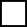
 Research Coordinator
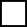
 Principal Investigator

Subject Meets Eligibility Criteria for Study?

Yes
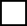
 No *If NO then DO NOT register subject in online database.*

*Destroy and discard this questionnaire within 90-days.*

PI Confirmation:

Print Name Signature

**If the subject is eligible** then the information in this questionnaire should be registered in the online database within 72 hours of confirmation of eligibility.

Submission Date: / /

Year Month Day
