# Supplementary material for: Metabolomics reveal distinct molecular pathways associated with future risk of Crohn’s Disease
Source: Gut Microbes. 2025 Sep 5;17(1):2546998. doi: 10.1080/19490976.2025.2546998 (PMC12416195; doi:10.1080/19490976.2025.2546998)
Supplement: Supplementary_Note_3.docx [file KGMI_A_2546998_SM8950.docx]

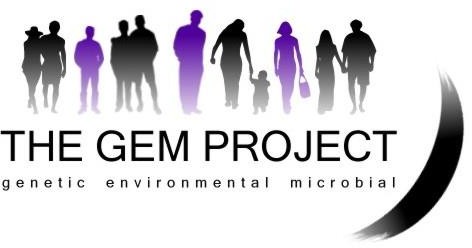


Document # 6

Confirmation of New Crohn’s Disease

| **Subject ID:** | – – | |
| --- | --- | --- |
| **Confirmation Visit Date:** | / /  Year Month Day |  |
| **Follow-up Date:** | / / | **Time:** |
|  | Year Month Day |  |

| Sample Type | Barcode Label |  |
| --- | --- | --- |
| **Urine** | (Affix label here) | Volume (ml) |
| **Stool** | (Affix label here) |  |
| **Blood** | (Affix label here) |  |

To be completed by the Research Assistant or Research Coordinator.

This form contains confidential and private personal health information and must be kept in the secure locked office of the Regional Coordinator.

Part 1 – Disease Information

| Confirmed Dx of Crohn’s Disease: | Yes No | Age at Diagnosis: |
| --- | --- | --- |
| Montreal Classification Available: | Yes No |  |
| Approx. Date of Disease Onset: | / / | *Partial date is permitted* |
|  | Year Month Day |  |
| Date of Diagnosis: | / / | *Partial date is permitted* |
|  | Year Month Day |  |
| Date of Last Clinical Encounter: | / / | *Partial date is permitted* |
|  | Year Month Day |  |
| Chart Review: |  |  |
| Family Physician: | Yes No N/A | Initial/Sign if done: |
| Specialist Physician: | Yes No N/A | Initial/Sign if done: |
| Hospital Chart: | Yes No N/A | Initial/Sign if done: |
| Have new medications been started? | Yes No | *List Medications* |
|  |  |  |
| Have new treatments been started? | Yes No | *List Treatments* |
| **Montreal Classification:** |  | *(e.g. A2-L2-B2 see reference below)* |
| Extra-intestinal Symptoms (free text) |  |  |
| Surgery (free text) |  |  |

| Age at Diagnosis (A) A1 (below 16 yrs)  A2 (between 17 and 40 yrs)  A3 (above 40 yrs) | Disease Location (L) L1 (Ileal)  L2 (Colonic)  L3 (Ileocolonic)  L4 (isolated upper disease*) | Behaviour (B)  B1 (non-stricturing, non-penetrating)  B2 (Stricturing)  B3 (Penetrating)  P (Perianal disease modifier**) |
| --- | --- | --- |

* L4 is a modifier that can be added to L1-L3 when concomitant upper GI disease is present.

** P is added to B1-B3 when concomitant perianal disease is present.

| Reports Available (check all that apply) | | Date (year/month/day) | CD Diagnosis Confirmed? |
| --- | --- | --- | --- |
| Endoscopy: | Yes No Unk. | / / | Yes No N/A |
| Histology: | Yes No Unk. | / / | Yes No N/A |
| Radiology: | Yes No Unk. | / / | Yes No N/A |

Part 2 – Antibiotics

| 1. Are you currently taking antibiotics or have you taken antibiotics in the past 30 days? | Yes No | *IF YES follow-up with subject 30 days after the last dose of antibiotics* |
| --- | --- | --- |
| 2. Have you taken antibiotics in the past 3 months? | Yes No | *If NO go to Q.4* |
| 3. If yes, which antibiotic(s)? | 1. |  |
|  | 2. |  |
| 4. Have you taken antibiotics in the past 6 months? | Yes No | *If NO go to Q.6* |
| 5. If yes, which antibiotic(s)? | 1. |  |
|  | 2. |  |
| 6. Have you taken antibiotics in the past 12 months? | Yes No |  |
| 7. If yes, which antibiotic(s)? | 1. |  |
|  | 2. |  |

Part 3 – Administrative

| Completed On: | / /  Year Month Day |  |
| --- | --- | --- |
| Completed by: |  |  |
|  | Print Name | Signature |
| Designation: | Research Coordinator Principal Investigator | |
| PI Confirmation: |  |  |
|  | Print Name | Signature |

The information on this form should be registered in the online database within 72 hours of completion. Please record the date of completion and the SUBJECT ID on all pages.

| Submission Date: | / /  Year Month Day |
| --- | --- |
